# Spatio-temporal analysis identifies marine mammal stranding hotspots along the Indian coastline

**DOI:** 10.1038/s41598-022-06156-0

**Published:** 2022-03-08

**Authors:** Sohini Dudhat, Anant Pande, Aditi Nair, Indranil Mondal, Mridula Srinivasan, Kuppusamy Sivakumar

**Affiliations:** 1 Department of Endangered Species Management, Wildlife Institute of India, Chandrabani, Dehradun, Uttarakhand 248001 India; 2 Marine Mammal and Turtle Division, Southeast Fisheries Science Center, National Marine Fisheries Service, 75 Virginia Beach Drive, Miami, FL USA

**Keywords:** Biodiversity, Conservation biology

## Abstract

Marine mammal strandings provide vital information on species’ life histories, population health and status of marine ecosystems. Opportunistic reporting of strandings also serve as a powerful low-cost tool for monitoring these elusive mammals. We collated data over ~ 270 years available through various open access databases, reports and publications. Annual strandings along the Indian coast (mean = 11.25 ± SE 9.1) increased in the last two years of the study (2015–2017, mean = 27.66 ± SE 8.5 strandings /year). We found that stranding events spike during June—September along the west coast and during December–January along the east coast. We identified several sections of the coastline, such as Mumbai (0.38 strandings/km), Kozhikode (0.28 strandings/km),  Tuticorin (0.4 strandings/km), Rameswaram (1.82 strandings/km), Chennai (0.32 strandings/km) and Bhubaneshwar (0.26 strandings/km) with a higher number of stranded animals reported. Emerging Hotspot Analysis located new and consecutive hotspots along the north-west coast, and sporadic hotspots along the south-east coast. We recommend establishing regional stranding response centres at the identified hotspots coordinated by a National Stranding Centre with adequately trained personnel and central funding support. Regular stranding response training programs for field veterinarians, and frontline personnel of State Forest Departments near stranding hotspots would provide an improved understanding of marine mammal health and threats in Indian waters. Further, the suggested National Stranding Centre needs to maintain a ‘National Stranding Database’ for long-term marine mammal conservation planning in India.

## Introduction

Understanding marine mammal stranding patterns are a useful and cost-effective way to obtain information on endemic marine mammal species in different parts of the world^1–4^. Stranding data provide crucial information on species life histories^5^, changes in population sizes inferred from stranding rates^6^, human-caused and natural threats to marine mammals, impact of anthropogenic activities on marine ecosystems and overall ocean health^6,7^. Marine mammal strandings occur due to complex interplay of ecological factors including various biological (diseases, parasitism-^8,9^; hearing impairments^10^); environmental (unusual tides, electrical storms^11^ ; cyclones-^12^ ; geomagnetic anomalies, echolocation distortion^13^) and anthropogenic factors (vessel strikes–^14^; entanglement in fisheries gear–^5,15,16^; noise pollution due to dredging, oil drilling, naval exercises^17–19^ and marine pollution^20,21^.

Marine mammal foraging areas often overlap with fisheries^22,23^ resulting in accidental entanglement, injuries or mortalities^24,25^. Shipping lanes intersect with movement corridors leading to collisions^26–28^ and noise pollution can cause ‘auditory masking’ and permanent or temporary acoustic injuries^29,30^ . Physical factors such as coastal topography (e.g. Cape Cod in Massachusetts, Golden Bay in New Zealand^1,31,32^), near shore surface currents, local wind patterns^33^ may cause geographic clustering of stranding events^13^. Factors such as gently sloping beaches, also known as ‘acoustic dead zones,’ distort acoustic signals of cetaceans, confounding navigation and leading to stranding^13^. Given these myriad factors, exact causes of strandings are challenging to establish and conclusive evidence is lacking in most instances. Further, only a fraction of the animals may show up on the beach alive or dead. A vast majority may die at sea and thus, remain undocumented^34–36^.

In India, an extensive coastline (~ 8000 km) and large Exclusive Economic Zone (EEZ, area ~ 2,305,143 km^2^) makes it arduous to document marine mammal strandings. Thirty-one species of marine mammals are estimated to exist in Indian EEZ (MMRCNI, http://www.marinemammals.in/). In the absence of scientific data on marine mammals along the Indian coast, primarily due to lack of broad-scale visual and acoustic surveys to estimate their population abundance, marine mammal stranding events can be a cost-effective and useful substitute to obtain data on local marine mammal occurrence patterns and potential threats.

Systematic recording of these events can help relevant government and non-government authorities to strategically invest resources to establish stranding response networks in key areas along the coast. Additionally, vital species-specific biological data from stranded specimens (live and dead) could help scientists and managers ascertain potential threats affecting local marine mammal populations and recommend appropriate mitigation measures. Given this context, we utilize publicly available data and data from systematic research surveys to identify stranding hotspots in India. We undertake a comprehensive analysis of marine mammal occurrence data to a) evaluate general trends in marine mammal occurrence; b) analyse long-term temporal (seasonal and annual) and spatial (along coasts, islands) patterns of marine mammal strandings; and c) and identify spatio-temporal hotspots for marine mammal stranding events along the Indian coastline. We consider our results to facilitate surveillance in key hotspots, develop early warning systems and inform marine mammal conservation strategies in the country.

## Results

Our compiled dataset consisted of 1674 records of marine mammal records after removing duplicate reports. It included 660 reports of sightings, 59 reports of induced mortalities or hunting records, 240 reports of incidental mortalities, 632 unique stranding records (live / dead), and 83 records which could not be categorised because of incomplete information.

### Sightings

**Figure 1:**
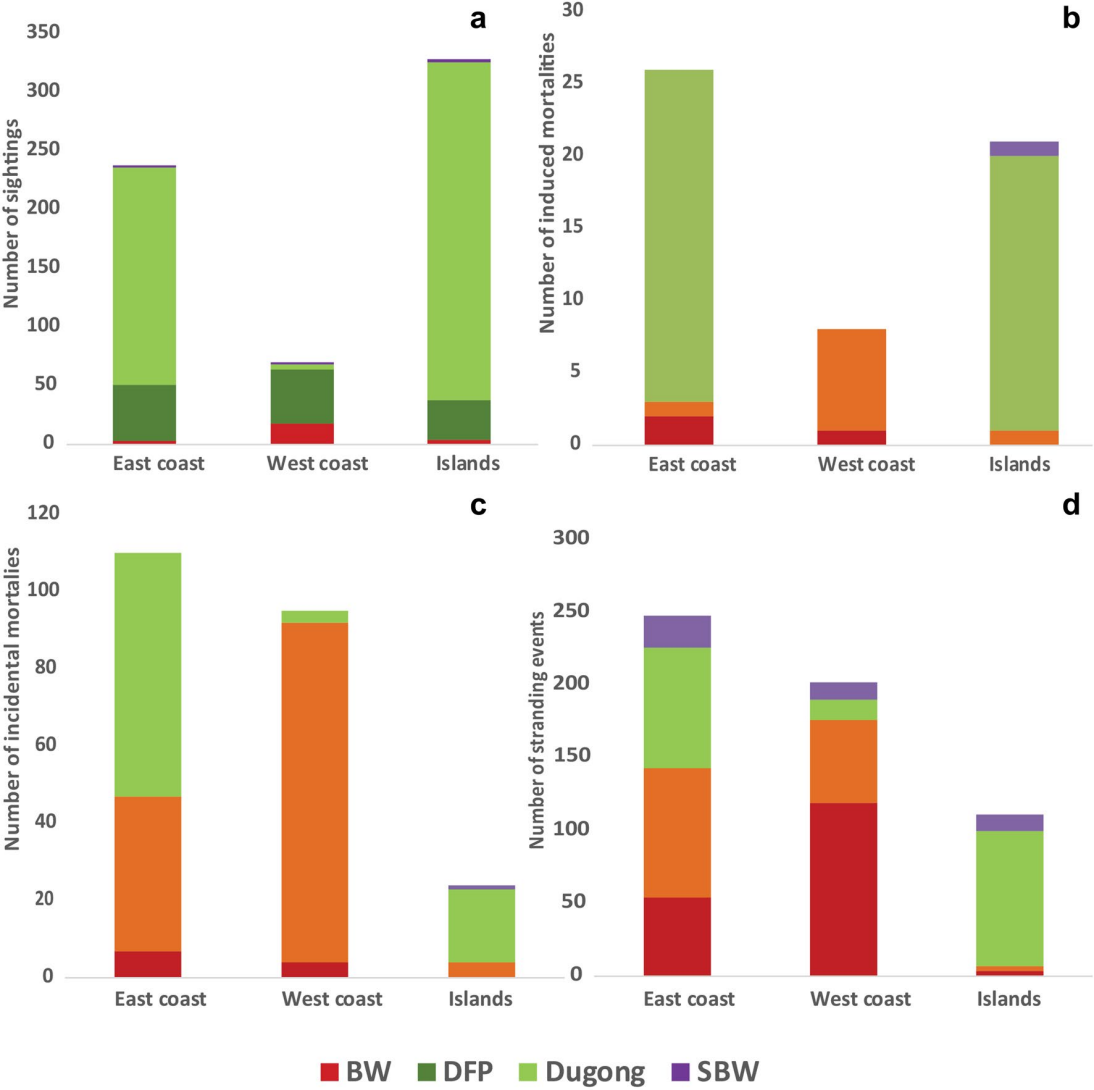
Marine mammal records obtained from data compiled between years 1748 – 2017 along the east coast, west coast and the islands of India for the groups i.e., baleen whales (BW), dolphins and finless porpoise (DFP), sperm and beaked whales (SBW) and dugongs, given as color-coded stacked bars where (**a**) sighting records—records where live animals were sighted (**b**) induced mortalities—records where animals were reported hunted or killed or were driven ashore, (**c**) incidental mortalities—records where animals were found dead after entanglement in fishing nets or being struck by vessels and (**d**) stranding records—records where dead or live animals were found washed ashore, or floating near shore or stranded alive and were attempted for rescue.

**Figure 2:**
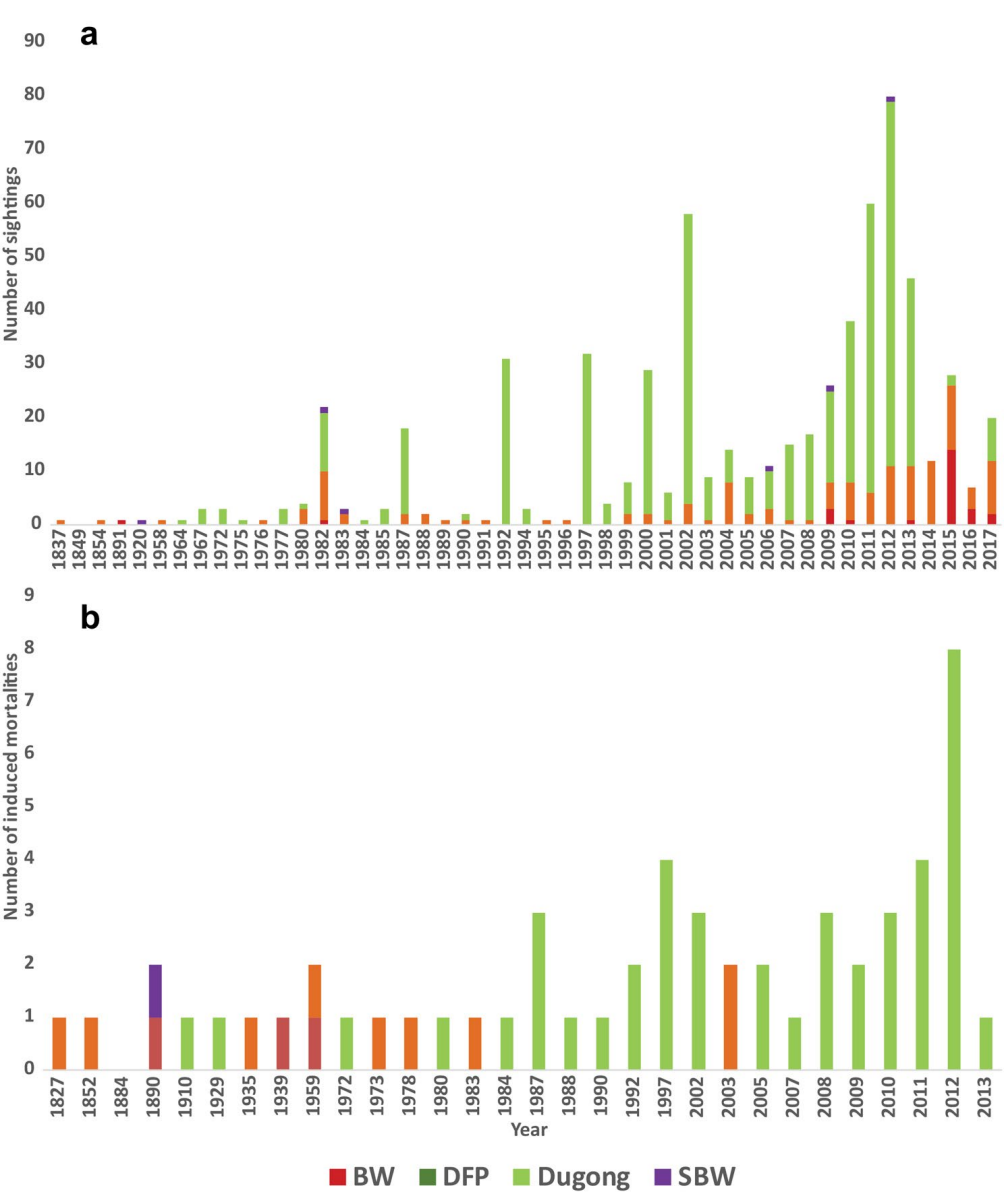

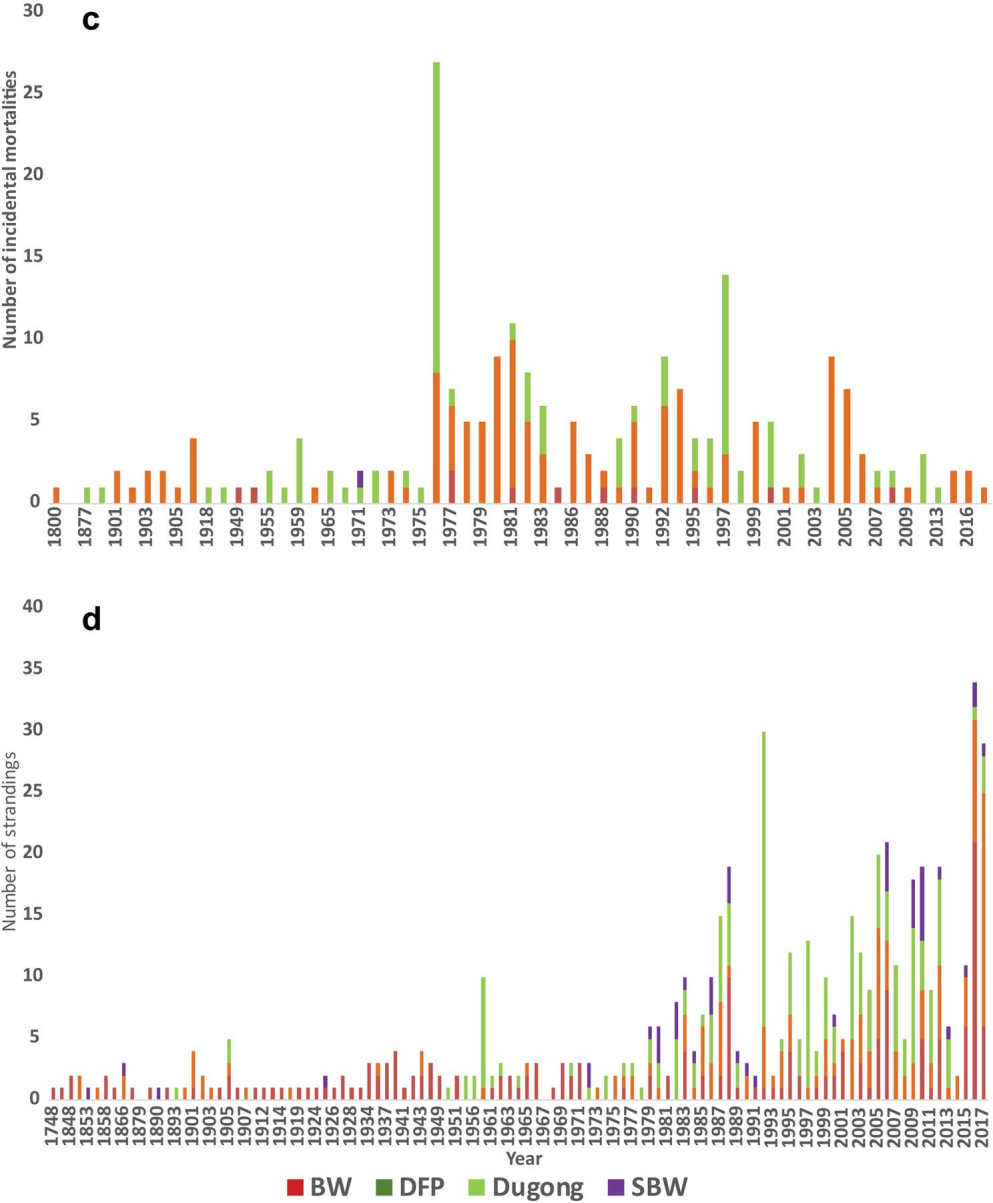
Marine mammal records obtained every year from the data compiled between years 1748–2017 along Indian coastline given as cumulative numbers for each group i.e., baleen whales (BW), dolphins and finless porpoise (DFP), sperm and beaked whales (SBW) and dugongs, as color-coded stacked bars, where (**a**) sighting records—records where live animals were sighted (**b**) induced mortalities—records where animals were reported hunted or killed or were driven ashore, (**c**) incidental mortalities—records where animals were found dead after entanglement in fishing nets or being struck by vessels and (**d**) stranding records—records where dead or live animals were found washed ashore, or floating near shore or stranded alive and were attempted for rescue.

A total of 660 opportunistic sightings (number of individuals, *n*_*i*_ = 3299) were recorded throughout the Indian coastline between 1748 and 2017 (Fig. 1a, 2a, 3a). Sighting data on the east coast (species = 18, *n*_*i*_ = 1105) was mostly restricted to Odisha and Tamil Nadu (representing 97% of total east coast sightings). On the west coast (*n*_*i* =_ 1297), Maharashtra (*n*_*i*_ = 549), Gujarat (*n*_*i*_ = 248) and Karnataka (*n*_*i*_ = 307) contributed to highest sighting records (representing 85% of total west coast sightings). Sightings from the islands also contributed to 24.85% of the dataset (Andaman & Nicobar Islands = 24.37%, Lakshadweep = 0.48%). Highest incidence of sightings was for DFP (*n*_*i*_ = 1894) followed by dugongs (*n*_*i*_ = 959), BW (*n*_*i*_ = 58) and SBW (*n*_*i*_ = 17).

**Figure 3:**
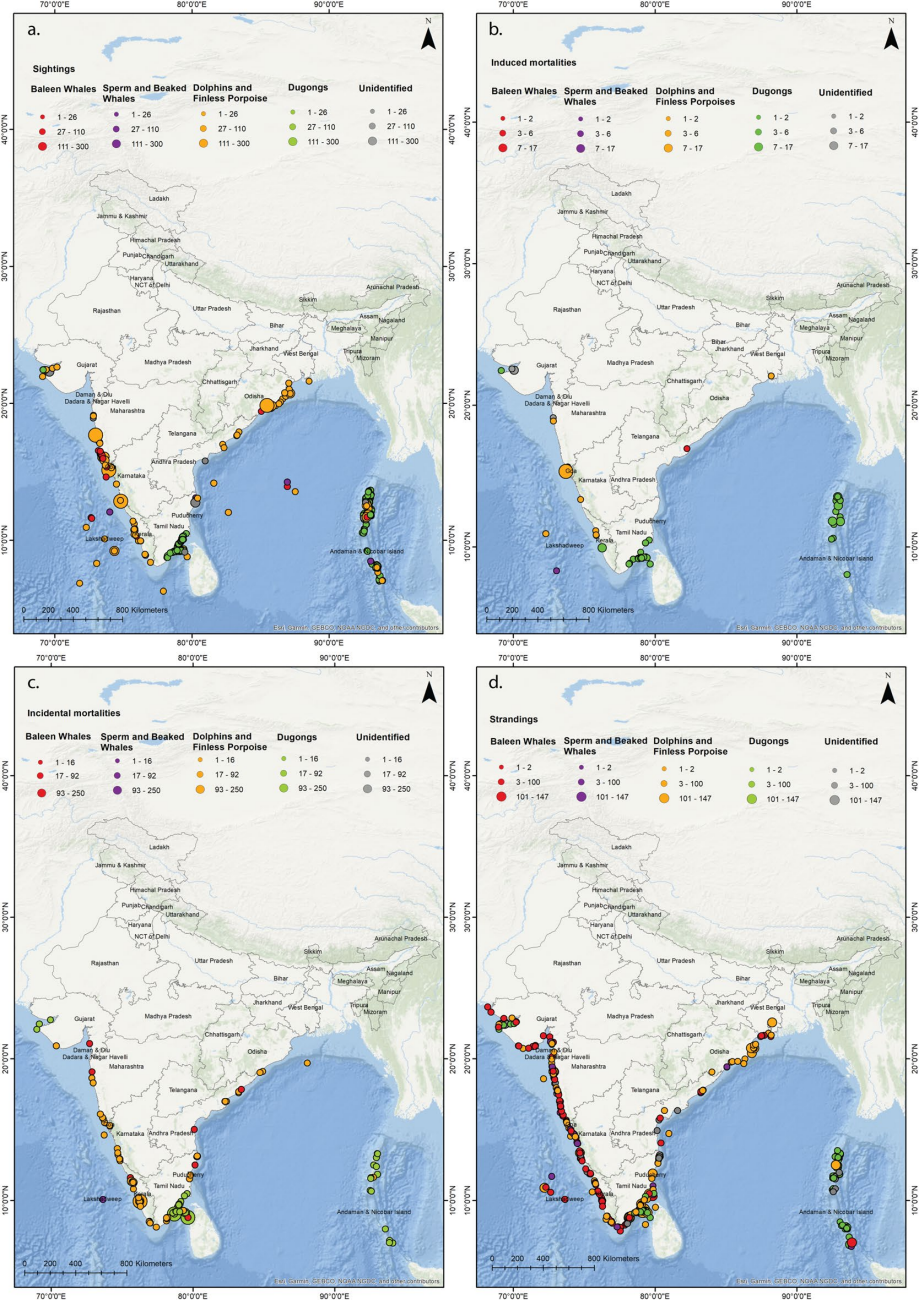
Bubble plots showing distribution of marine mammal records obtained from data compiled between years 1748–2017 along the Indian coastline for each group i.e., baleen whales (BW), dolphins and finless porpoise (DFP), sperm and beaked whales (SBW) and dugongs, as color-coded stacked bars, where (**a**) sighting—records where live animals were sighted (**b**) induced mortalities—records where animals were reported hunted or killed or were driven ashore, (**c**) incidental mortalities—records where animals were found dead after entanglement in fishing nets or being struck by vessels and (**d**) strandings—records where dead or live animals were found washed ashore, or floating near shore or stranded alive and were attempted for rescue. Size of the bubble indicates number of individuals. These maps were created using ArcGIS 10.5 (https://desktop.arcgis.com/en/arcmap/10.3/map/working-with-layers/about-symbolizing-layers-to-represent-quantity.htm).

### Induced mortalities

A total of 59 incidences (*n*_*i*_ = 102) were recorded of marine mammals being hunted/ captured between the years 1748–2017 (Fig. 1b, 2b, 3b). The total number of animals hunted/ captured deliberately is similar along east coast (*n*_*i*_ = 33), west coast (*n*_*i*_ = 29) and islands (*n*_*i*_ = 36). Out of all marine mammal species, 90% of the animals hunted at the east coast were dugong *D. dugon* (*n*_*i*_ = 30, all from Tamil Nadu). On the west coast, records of hunting incidences of finless porpoise *Neophocaena phocaenoides* were highest (79% of total records on west coast, Goa *n*_*i*_ = 17, Kerala *n*_*i*_ = 4, Karnataka and Maharashtra *n*_*i*_ = 1). In the islands (i.e., Andaman and Nicobar Islands), 94% of the hunting records were of dugongs (*n*_*i*_ = 34).

### Incidental mortalities

A total of 240 net entanglements (*n*_*i*_ = 1356) were reported along the Indian coast between the years 1748 and 2017 (Fig. 1c, 2c, 3c). Similar counts of individuals entangled along east (*n*_*i*_ = 670) and west coast (*n*_*i*_ = 654) were obtained with low reporting from the islands (*n*_*i*_ = 26). Fourteen species were reported entangled from both east and west coast with only 4 species recorded from the islands. *D. dugon* was found to be most frequently entangled along the east coast (63 incidences, *n*_*i*_ = 594, contributing to 56% of the total numbers on east coast), followed by *Tursiops sp.* (11 incidences, *n*_*i*_ = 14, 9% of the east coast dataset). On the west coast, *Tursiops sp.* was the most frequently entangled (18 incidences, *n*_*i*_ = 117, contributing to 18% of the west coast dataset), followed by *N. phocaenoides* (17 incidences, *n*_*i*_ = 34, contributing to 17% of the dataset). The total number of DFP being entangled from west coast (*n*_*i*_ = 623) were higher than east coast (*n*_*i*_ = 68). More dugong individuals were entangled along east coast (i.e., from Tamil Nadu; *n*_*i*_ = 594) as compared to the west coast (i.e., Gujarat; *n*_*i*_ = 3) and Islands (i.e., Andaman and Nicobar; *n*_*i*_ = 19). *D. dugon* was the most frequently entangled species in the islands (19 incidences, *n*_*i*_ = 19, contributing to 79% of the total numbers in islands dataset) followed by false killer whale *Pseudorca crassidens* (3 incidences, *n*_*i*_ = 5, contributing to 12% of the islands dataset). Very few BW or SBW (11 incidences, *n*_*i*_ = 11) were recorded accidently entangled throughout the Indian coastline.

### Strandings

Marine mammals stranding reports consisted of 91.93% dead (*n*_*i*_ = 581) and 8.07% live strandings (*n*_*i*_ = 51) (Figs. 1d, 2d, 3d). Considering mass strandings as strandings with *n*_*i*_ > 2 (excluding mother and calf;^33,34^), 8.5% of all reports were mass strandings (21 strandings, *n*_*i*_ = 1054). Most of the records did not have information about the sex of the stranded animal (83%), the age class (88%) or the state of decomposition of the carcass (53%). Highest strandings were reported of dugongs (strandings = 190, *n*_*i*_ = 228), followed by BW (strandings = 178, *n*_*i*_ =  = 190), DFP (strandings = 157, *n*_*i*_ =  = 552) and SBW (strandings = 47, individuals = 48). There were 54 incidences (*n*_*i*_ = 54, 9% of total stranding data) where the animal was not identified reliably to include in either of the groups.

Species composition and frequencies of strandings were different on east coast, west coast and in the islands (Fig. 1, Table 1). Twenty-two species were reported as stranded on the east coast with *D. dugon* as the most frequently stranded species (83 incidences, *n*_*i*_ = 107, ~ 29% of all records), followed by Indo-Pacific humpback dolphin *Sousa chinensis*, (31 incidences, *n*_*i*_ = 108, ~ 10% of all records). On the west coast, out of 20 species reported as stranded, *Balaenoptera musculus* was most frequent (28 incidences, *n*_*i*_ = 29, ~ 12% of all records) followed by *N. phocaenoides* (23 incidences, ni = 39, ~ 10% of all records). In the islands, 13 species were reported as stranded, *D. dugon* (93 incidences, *n*_*i*_ = 102, contributing to 77% of the total animals found on the islands) followed by strandings of sperm whale *Physeter macrocephalus* (8 incidences, *n*_*i*_ = 8, contributing to 6% of the data; Table 1).

- **a. Baleen whales**

**Table 1:** Number of stranding events reported for marine mammals between 1748–2017 in India from the east coast, the west coast and Lakshadweep and Andaman & Nicobar archipelagos.

Species	East coast		West coast		Islands	
	Dead strandings (n_i_)	Live strandings (n_i_)	Dead strandings (n_i_)	Live strandings (n_i_)	Dead strandings (n_i_)	Live strandings (n_i_)
**Baleen Whales (BW)**						
Balaenoptera acutorostrata*		1 (1)			1 (1)	
Balaenoptera borealis*	11 (11)	1 (1)	3 (3)			
*Balaenoptera edeni*	6 (6)	1 (1)	18 (21)		2 (2)	
*Balaenoptera musculus*	7 (7)	1 (1)	22 (23)	6 (6)	1 (1)	
Balaenoptera physalus*	3 (3)	1 (1)	8 (8)	1 (1)		
*Balaenoptera sp.*	21 (27)		55 (57)	1 (1)		
*Megaptera novaeangliae*	1 (1)		5 (5)	1 (1)		
**Sperm and beaked Whales (SBW)**						
*Kogia breviceps*	1 (1)		1 (2)			
*Kogia sima*	2 (2)		1 (1)	2 (2)	1 (1)	
*Mesoplodon densirostris*					1 (1)	
*Mesoplodon pacificus*					2 (2)	
*Physeter macrocephalus*	19 (19)	1 (1)	5 (5)	1 (1)	7 (7)	1 (1)
*Ziphius cavirostris*			2 (2)		1 (1)	
**Dolphins and Finless porpoise (DFP)**						
*Delphinus capensis*	2 (43)		1 (1)			
*Globicephala macrorhynchus*	3 (27)	1 (147)				1 (40)
*Grampus griseus*	2 (2)				1 (1)	
*Orcaella brevirostris*	6 (6)	1 (1)				
*Orcinus orca*			1 (1)			
*Pseudorca crassidens*	4 (4)		5 (8)			
*Sousa chinensis*	30 (102)	1 (6)	10 (11)			
*Sousa plumbea*			16 (16)			
*Stenella attenuata*	2 (13)					
*Stenella longirostris*	7 (22)	2 (2)	1 (3)			
*Steno bredanensis*			2 (2)			
*Tursiops aduncus*	7 (9)		2 (2)			
*Tursiops truncatus*	5 (5)					
*Neophocaena phocaenoides*	20 (29)		21 (37)	2 (2)	1 (1)	
**Dugongs**						
*Dugong dugon*	72 (73)	11 (34)	13 (18)	1 (1)	67 (72)	2 (2)
**Total**	**231 (412)**	**22 (196)**	**192 (226)**	**15 (15)**	**84 (98)**	**4 (43)**

* Possible misidentifications.

A total of 178 BW strandings (*n*_*i*_ = 190) were reported. Most species were unidentified (east coast *n*_*i*=_ 27, west coast *n*_*i*_ = 58, islands *n*_*i*_ = 4; i.e., 47% of the data). Identified strandings comprised of 6 species (see Table 1), some of which were later found to be misidentification (no confirmed evidence for common Minke Whale *Balaenoptera acutorostrata,* Sei Whale *Balaenoptera borealis* and Fin Whale *Balaenoptera physalus* from Indian waters; MMRCNI, 2018). Higher number of strandings occurred on the west coast (*n*_*i*_ = 126), as compared to east coast (*n*_*i*_ = 60). The east and west coast reported all six species of BW, whereas only three species stranded on the islands. *B. borealis* (misidentified) was the most stranded species across the east coast (12 incidences, *n*_*i*_ = 12, contributing to 11% of the data) whereas blue whale *Balaenoptera musculus* was the most frequent across the west coast (28 incidences, *n*_*i*_ = 29, contributing to 11% of the data). Baleen whale strandings were rare in the islands (4 incidences, *n*_*i*_ = 4).

- **b. Sperm and beaked whales**

Forty-seven SBW strandings (*n*_*i*_ = 48) were reported along the Indian coast. More SBW stranded on the east coast (*n*_*i*_ = 23) as compared to the west coast (*n*_*i*_ = 13) and the islands (*n*_*i*_ = 12). *P. macrocephalus* was most frequently reported (70% of all SBW records, east coast *n*_*i*_ = 20, west coast *n*_*i*_ = 6, islands *n*_*i*_ = 8).

- **c. Dolphins and finless porpoise**

There were 157 strandings (*n*_*i*_ =552) of DFP belonging to 14 species. Twenty-one of these events were mass strandings (*n*_*i*_ > 2). The largest mass stranding event (*n*_*i*_ = 147) occurred of short-finned pilot whale *Globicephala macrorhynchus* along the west coast (Tamil Nadu). Higher number of DFP strandings were recorded from east coast (*n*_*i*_ = 418) as compared to west coast (*n*_*i*_ = 83) and the islands (*n*_*i*_ = 51; Table 1). East coast received a higher diversity of stranded DFP (number of species = 11) as compared to west coasts (number of species = 9) and the islands (number of species = 3). *S. chinensis* was the most frequently stranded species along the east coast (31 incidences, *n*_*i*_ = 108, contributing to 33% of the data) whereas *N. phocaenoides* was the most frequent along the west coast (23 incidences, *n*_*i*_ = 39, contributing to 37% of the data; Table 1).

- **d. Dugongs**

The current distribution of dugongs in India is in the shallow coastal waters of Gujarat, Tamil Nadu and Andaman & Nicobar Islands^37,38^. There are 190 stranding events recorded between the years 1893 and 2017. The highest number of stranded dugongs were recorded from Tamil Nadu (*n*_*i*_ = 107) closely followed by Andaman and Nicobar Islands (*n*_*i*_ = 102) and few records from Gujarat (*n*_*i*_ = 19).

### Temporal stranding patterns

**Figure 4:**
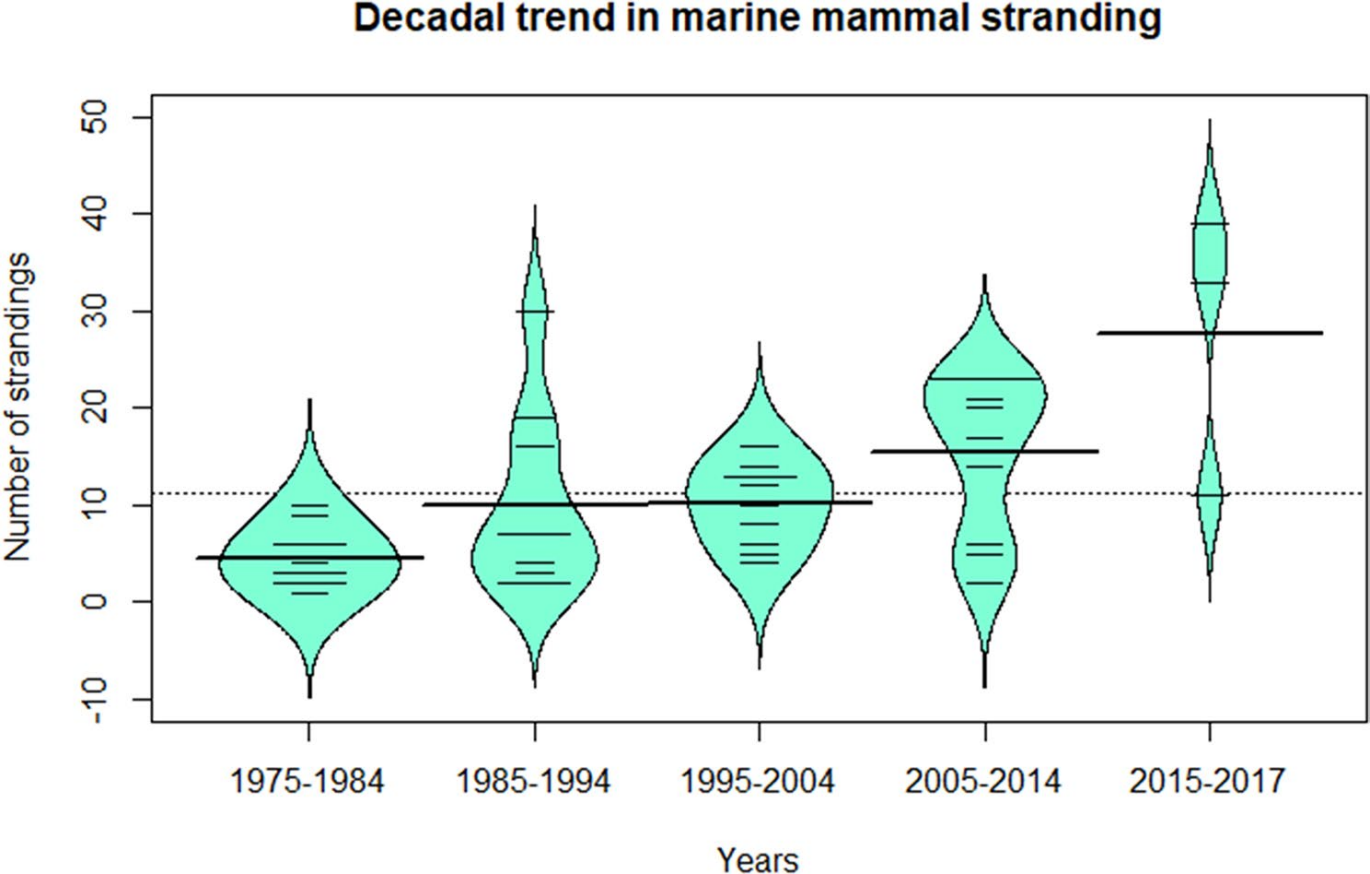
A beanplot of decadal trends in marine mammal stranding in India from data compiled between years 1975–2017. Data prior to 1975 was discontinuous over the years to be considered for decadal trends. The data for last decade considered here includes only two years (2015–17) where increased reporting is evident. The bold horizontal lines indicate the mean number of strandings in each decade whereas the smaller horizontal lines indicate stranding numbers recorded for each year within the decade.

Our analysis of temporal trends for the last 42 years (1975–2017) showed that the mean number of strandings along the Indian coast was 11.25 ± SE 1.39 / year. The number of stranding reports show an increasing trend for two decades after 1975, dropping between 1995 and 2004. We observed a distinct rise in strandings post 2005 (18.23 ± SE 2.98 / year) with the highest reports from 2015–17 (27.66 ± SE 8.51/year) (Fig. 4).

- **a. Baleen whales**

**Figure 5:**
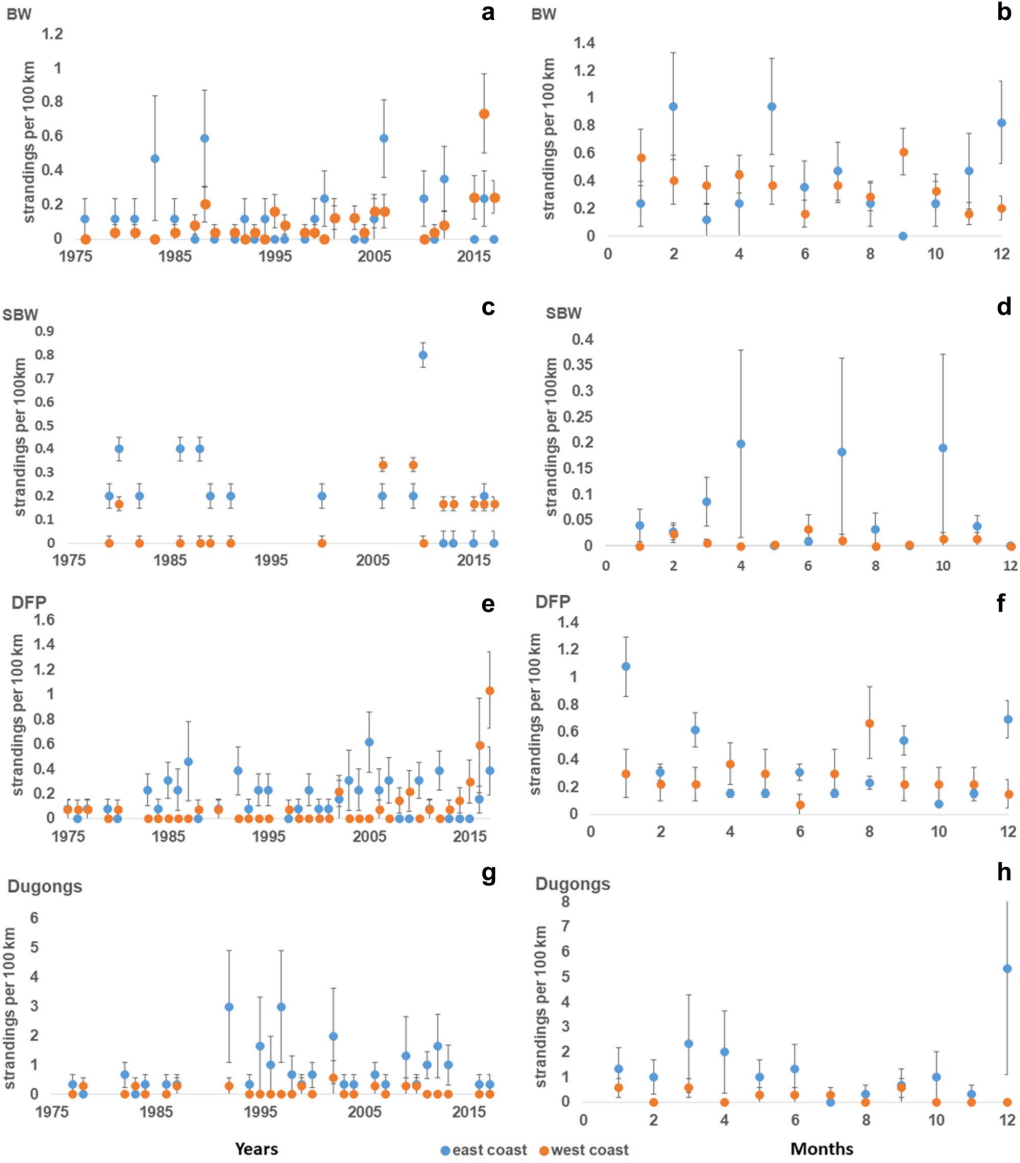
Temporal patterns (annual and monthly stranding rates / 100 km of coastline) in strandings of marine mammal records obtained from data compiled between years 1975–2017 along east and west coast of India for each group where (**a**) annual stranding rate and (**b**) monthly stranding rate for baleen whales (BW); (**c**) annual stranding rate and (**d**) monthly stranding rate for dolphins and finless porpoise (DFP); (**e**) annual stranding rate and (**f**) monthly stranding rate for sperm and beaked whales (SBW) and (**g**) annual stranding rate and (**h**) monthly stranding rate for dugongs.

On the west coast, mean stranding rate throughout the years (1975–2017) was 0.0010 ± SE 0.0014 strandings/km, and a steady rise was observed in rate of reported strandings after 2010. A seasonal trend was observed as well, with a peak in the month of September (*s*_*r*_ = 0.0061 ± SE 0.0016 strandings/km), i.e., towards the end of monsoon season, and lowest strandings were recorded in the month of June (*s*_*r*_ = 0.0016 ± SE 0.006 strandings/ km) (Fig. 5).

The mean stranding rate of BW on the east coast through 1975–2017 was 0.0013 ± SE 0.0017 strandings/km, but no specific trends were observed according to years or seasons. Stranding rates of BW did not differ between east and west coast (Mann–Whitney U test, U = 390, U standardized = -0.025, *p* value > 0.05).

- **b. Sperm and beaked whales**

The stranding rates of SBW differed significantly along both the coasts (Mann Whitney U test, U = 192, U standardized = 0.0, *p* value < 0.05). The mean stranding rate of SBW on west coast was 0.0010 ± SE 0.0012 strandings/km, whereas on east coast was 0.0022 ± SE 0.002 strandings/km. The strandings do not show any specific patterns over the years. The stranding events of SBW are too low (n =  < 5 per month) along both the coasts so no seasonal patterns are observed (Fig. 5).

- **c. Dolphins and finless porpoise**

The stranding rates of DFP differed significantly along both the coasts (Mann Whitney U test, U = 1008, U standardized = 3.61, *p* value = 0.00). The mean stranding rates of DFP across the west coast was 0.0009 ± 0.0019 strandings/km, increasing after 2014 (Fig. 5).

A seasonal trend was observed with a definite rise during monsoon, with highest number of strandings recorded in August (*s*_*r*_ = 0.0066 ± SE 0.013 strandings/ km) (Fig. 5). Along the east coast, the mean stranding rate across the years was 0.0016 ± SE 0.0015 strandings/km. The number of strandings increase after November, post retreating monsoon season, with the highest number of strandings in January (stranding rate = 0.010 ± SE 0.018 strandings/ km) (Fig. 5).

- **d. Dugongs**

The stranding rates of dugongs show significant differences between Gujarat (west coast) and Tamil Nadu (east coast) (Mann Whitney U test, U = 681, U standardized = 5.65,* p *value =  < 0.0001). The rate of dugong strandings in Gujarat was 0.0010 ± SE 0.0016 strandings/km (n =  < 5) and therefore, difficult to comment on seasonal/ monthly patterns. The mean stranding rates through the years on the east coast (i.e., from Tamil Nadu) was 0.0083 ± SE 0.008 strandings/km and strandings were found to be the highest in December (*s*_*r*_ = 0.0533 ± SE 0.104 strandings/km) (Fig. 5).

### Spatial patterns

**Figure 6:**
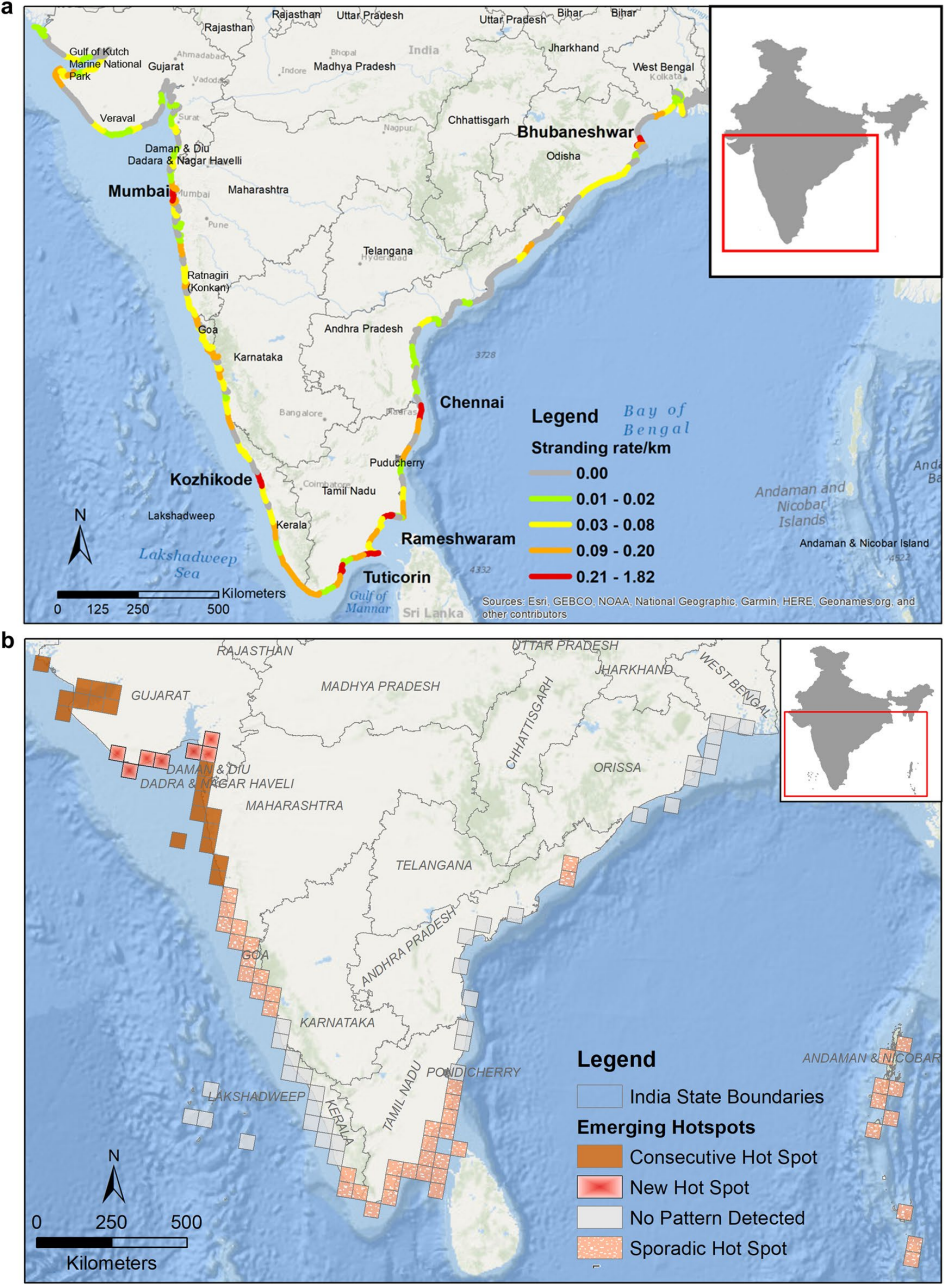

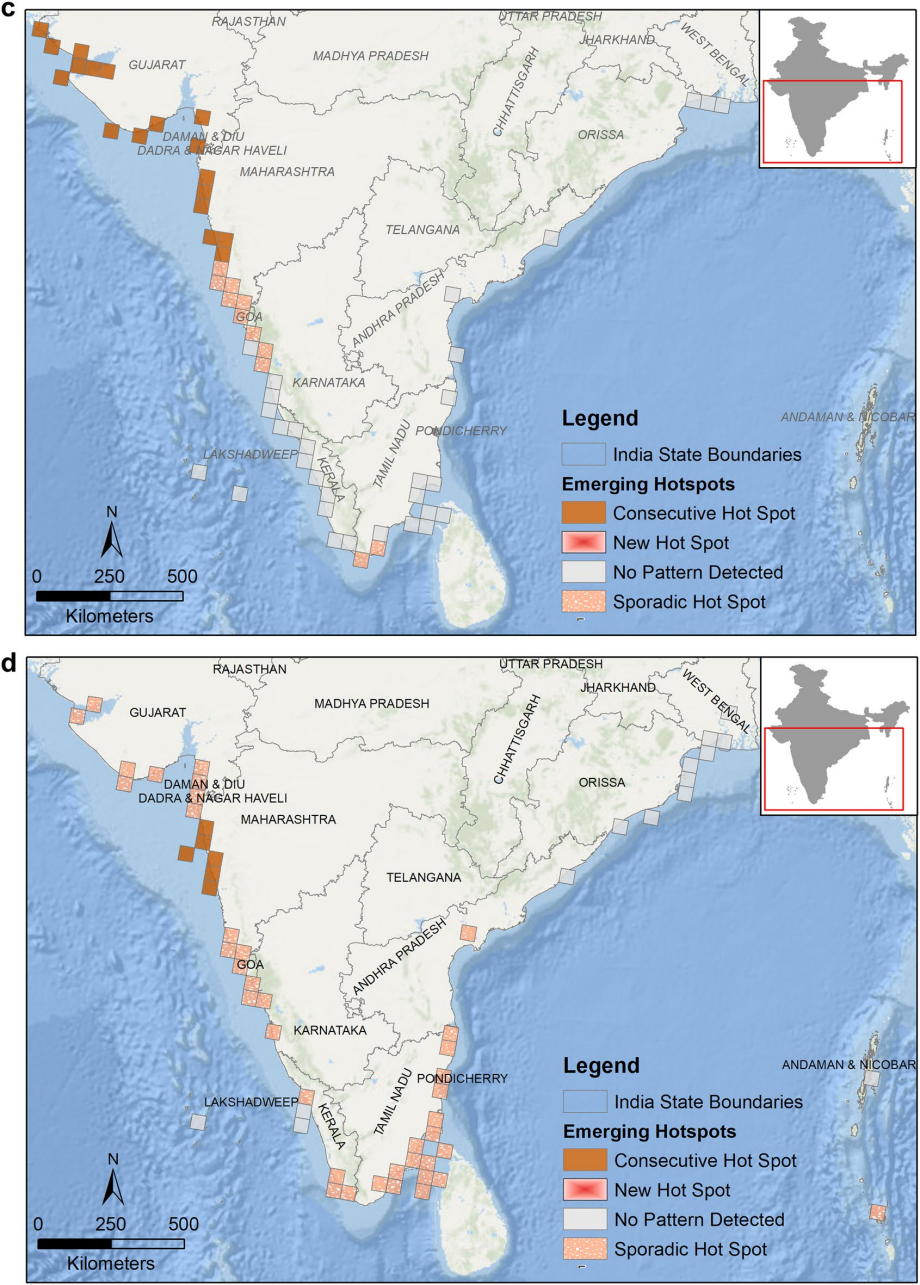
(**a**) Stranding rates (number of strandings/ km) of marine mammals calculated from data compiled between years 1748–2017 along the Indian coastline. High strandings rates (in red) are observed at sections near Mumbai, Kozhikode, Tuticorin, Chennai and Bhubaneshwar. This map was created using ArcGIS 10.5 (https://desktop.arcgis.com/en/arcmap/latest/tools/analysis-toolbox/near.htm). (**b**) Emerging hotspots obtained from all marine mammal stranding records from data compiled between years 1748–2017 along the Indian coastline. This map was created using ArcGIS Pro 2.4.2 (https://pro.arcgis.com/en/pro-app/2.8/tool-reference/space-time-pattern-mining/emerginghotspots.htm). (**c**) Emerging hotspots obtained from all stranded baleen whale records from data compiled between years 1748–2017 along the Indian coastline. This map was created using ArcGIS Pro 2.4.2 (https://pro.arcgis.com/en/pro-app/2.8/tool-reference/space-time-pattern-mining/emerginghotspots.htm). (**d**) Emerging hotspots obtained from all stranded dolphins and finless porpoise records from data compiled between years 1748–2017 along the Indian coastline. This map was created using ArcGIS Pro 2.4.2 (https://pro.arcgis.com/en/pro-app/2.8/tool-reference/space-time-pattern-mining/emerginghotspots.htm).

We observed higher strandings near Mumbai (0.38 strandings/km), Kozhikode (0.28 strandings/km), Tuticorin (0.4 strandings/km), Rameswaram (1.82 strandings/km), Chennai (0.32 strandings/km) and Bhubaneshwar (0.26 strandings/km) (Fig. 6a). Even though the total strandings along east coast are more than twice that of west coast (refer Table 1), they are concentrated towards Tamil Nadu region rather than being spread out evenly along the coast. On the other hand, strandings on west coast are evenly spread out with each coastal section reporting strandings.

#### Emerging hotspot analysis

Only 68.67% of stranding events (*n*_*s*_ = 632) could be used for emerging hotspot analysis due to lack of information on the stranding event month. The analysis detected four emerging hotspot categories: no patterns, consecutive, sporadic, and new hotspots (see definition in Table 2).

**Table 2:** Emerging hotspot categories detected and their definitions.

Sr. no	Symbol	Category detected	Definition
1		No pattern detected	Does not fall into any of the hot or cold spot patterns defined below
2		New hotspot	An area which has become statistically significant hotspot in final time step, i.e. for the years 2016 and/or 2017
3		Consecutive hotspot	An area which has been statistically significant hotspot for a considerable timeframe before the final time-step, i.e., in the years 2010 onwards
4		Sporadic hotspot	An area which has been a hotspot on and off throughout the time period. Less than 90% of the data (37 years out of the 42) have been statistically significant hot spots and none of the time-step intervals have been statistically significant cold spots

Along the west coast, the southern region of Gujarat, near Veraval (district Gir Somnath) and the coast of Surat emerged as new hotspots (Fig. 6b). It implies that these regions were never a hotspot but the frequent strandings in the last time step, i.e., in 2017, being statistically significant, highlight them as new hotspots (see supplementary material).

The area around Gulf of Kutch Marine National Park, Gujarat, and most of the Konkan coast, except Ratnagiri, Maharashtra reported several strandings in recent years, emerging as consecutive hotspot (Table 2, Fig. 6b). Further, the northern Karnataka coast, south of Kerala and Tamil Nadu, Vishakhapatnam and Andaman and Nicobar Islands are sporadic hotspots. Strandings in these regions were higher but temporally inconsistent (ESRI, ArcGIS Pro, 2016) thus making it difficult to be demarcated as hotspots. No significant pattern was detected near southern Karnataka, northern Kerala, Lakshadweep Islands, Pondicherry, Andhra Pradesh, Odisha, and West Bengal states.

- **a. Baleen whales**

The baleen whale strandings resulted in three patterns. The Gujarat and Maharashtra coasts emerge as consecutive hotspots, due to recent baleen whale strandings (i.e., 2015–2017). Northern part of Karnataka and Kanyakumari are sporadic hotspots, which means they have been hotspots for baleen whale strandings on and off throughout the study period (Fig. 6c). No pattern was detected from rest of the strandings of BW along the coast.

- **b. Dolphins and finless porpoise**

The strandings of DFP were sporadic throughout the west coast, except the region between Mumbai and Ratnagiri, which is a consecutive hotspot. No patterns were detected from strandings along north of Andhra Pradesh, Odisha, West Bengal, Lakshadweep, and Andaman Islands (Fig. 6d). Stranding records of SBW and dugongs were few (< 60 records) to detect any hotspots (ESRI, ArcGIS Pro version 2.4.2).

## Discussion

This study is the first attempt to use publicly available data on marine mammal strandings to identify stranding hotspots in India. The dataset used in the study was compiled from scientifically vetted databases, primary surveys, government reports and newspaper articles providing a comprehensive synthesis of long-term marine mammal stranding records in the country. Even though temporally discontinuous and lacking uniformity in reporting parameters, this dataset provides critical information for managing marine mammal strandings across the vast Indian coastline. Adding over 752 unique records to the existing MMRCNI database, this dataset helped us a) to highlight general patterns in marine mammal occurrence b) illustrate spatio-temporal patterns in strandings providing evidence for emerging hotspots and c) identify critical areas for developing a robust stranding response program along the Indian coastline.

Reports of marine mammal occurrence have steadily increased over the last two decades reflective of increased awareness about marine mammals, frequent reporting of strandings with enhanced internet connectivity in remote areas and dedicated survey effort along some coastal areas^39–41^. Maximum sighting reports used in this study were of *D. dugon* obtained through interview-based surveys of fisher communities at three sites of Gulf of Kutch (Gujarat), Palk Bay & Gulf of Mannar (Tamil Nadu) and Andaman & Nicobar Islands^42^ followed by those of *Sousa* sp. on the west coast^40,43,44^. Hunting or capture data identified *N. phocaenoides* on the west coast (from the years 1827–1983) and *D. dugon* on the east coast being hunted in the past as well as from records in the past ~ 100 years (records from 1910 to 2013). High hunting incidences from Tuticorin and Ramanathapuram districts of Tamil Nadu suggest a need for enhanced conservation awareness and intensive monitoring in these areas. Identical patterns in fishnet entanglement appear from both coasts with *D. dugon* being most frequently entangled on the Tamil Nadu coast and DFP being reported widely from all the west coast along with few reports of BW or SBW entanglements.

Most stranding events comprised of single individuals with little to no information on the condition of the carcass, gender identification or age class. Lack of systematic necropsies reflects in the dataset with several species’ misidentifications in the dataset. Media reporting is also often erroneous, which can further confuse species identification and stranding causes. High *D. dugon* strandings have been reported through focused surveys^42^ and formation of volunteer networks^45,46^ from isolated pockets of dugong distribution along the Indian coast. This absence of quality information collection on stranding events arises due to lack of appropriate facilities and trained personnel to report strandings and conduct necropsies throughout the Indian coastline. Further, most biological information is lost with a delay in reporting and/or misidentification at the stranding site indicative of weak coordination between responders, managers, and researchers for an appropriate stranding response^47^. Moreover, there is no dissemination of the results from necropsies or tissue analysis in peer-reviewed articles, technical reports or through any government portal or approved website.

Marine mammals migrate locally^48^ as well as long-distance^49^ in response to changes in habitat characteristics (such as sea surface temperature or prey movement) to breed or to find optimal foraging regions^50,51^. Long-term stranding datasets facilitate monitoring these movements, identifying novel breeding, or foraging habitats and decoding linkages between climate variability and shifting marine mammal distributions^51^. We observed a distinct rise in strandings over decadal time scales post 2005, with the highest frequency of reported strandings noted between 2015 and 17 (n_s_/year = 27.66, 13% of total strandings). This stranding rate is comparable to some well monitored coastlines (e.g. Chile, south Australia) of the world reporting high marine mammal strandings^52,53^. Among groups, BW strandings show a steady increase along west coast post 2010, peaks observed in September (i.e., post-monsoon) while lowest reports from June. No specific seasonal trends of BW were observed along east coast. SBW did not show any specific seasonal patterns with few records from either coast. DFP show an increase in strandings after 2014 along the west coast esp. during August (i.e., post-monsoon). Along the east coast, the DFP strandings rise after November peaking in January. For dugongs, numbers along west coast were too low to detect any seasonal patterns but along east coast (Tamil Nadu), the strandings peak in December. These patterns emerge from a multi-scale (coasts) and multi-species dataset illustrating the complexity, seasonality, varying anthropogenic pressure and reporting differences of the stranding events. Suitable management interventions and focused monitoring is required along coastal sections where higher strandings have been reported in these specific seasons to decipher the precise causes.

Globally, spatial patterns of marine mammal strandings point towards localised threats in certain regions^54^ e.g. use of high powered SONARs for bathymetric studies or for military use^55,56^, deep sea oil explorations^57^, negative interactions with fishing gears^58^, or vessel strikes near shipping ports or high tourism areas^26^. Our spatial analysis revealed that the geographical distribution of the strandings was not homogenous and division of the coastline into sections revealed the patterns on a finer scale (50 km). We found that the stranding rates on the sections of west coast oscillated between low to moderate, with few sections of the coasts (districts of Mumbai and Kozhikode) displaying high stranding rates. In contrast, the southern sections of the east coast had low to moderate strandings with higher rates from Tamil Nadu, whereas the coastline in the central and northern region had negligible stranding rates, with one or two exceptions.

Spatial stranding patterns of marine mammals in India coincide with monsoon winds which flow towards the landmass along the respective coast^59,60^. The monsoon season differs along both coasts as southwest winds bring rains to west coast in the months of June–September, whereas the east coast experiences retreating monsoonal winds from the north-eastern direction, between October–November^61^. This changes the ocean currents and sea surface temperature patterns during two distinct periods across the Indian subcontinent^59,60^. Post-mortem deposition of carcasses along certain sections of the coastline are thus governed by these environmental factors^1,33,51^. The observed patterns are thus possibly an artefact of seasonal changes in these hydrodynamic drivers and are likely to increase stranding events when the currents drive the animals towards the shore or reduce if the dead animals are driven away into deeper regions of the ocean. These results highlight the need of validated reporting of strandings along the Indian coastline to understand the effect of climatic drivers, which are likely to be highly informative over a longer time frame^62,63^. Increased strandings in certain seasons might also point to increased presence of a species in a region in particular season. Studies on blue whales in North Indian Ocean have pointed towards the fact that they forage in the upwelling zones along the southwest coast of India and Sri Lanka during the monsoons, and later disperse as the upwelling ceases after monsoon is over^64^. This could possibly explain the increased numbers of baleen whale strandings in the monsoons on west coast.

Increased stranding rates might relate to extreme climatic conditions^63,65^, oceanographic anomalies^32^, exposure to pollutants^66^ and other biological events (e.g. red tides, diseases, predation, carrying capacity, prey availability)^66–69^. Besides these, certain human activities such as near shore gill-netting, destructive fishing practices, effluent discharge, and intensive vessel traffic^26^ pose localised threats which increase strandings . We cannot discount the possibility that these events might result in varying patterns of strandings across finer or greater spatial scales than the one chosen for the current study. The patterns obtained in this study provide a baseline for researchers to investigate using data on species distribution, localised threats, and variations in physical oceanographic processes.

Despite the challenges and limitations of working with an opportunistic database and clustered reporting tendencies (more reporting from Tamil Nadu and ANI), our study has revealed strong spatiotemporal patterns with significant implications for both conservation biologists and biodiversity management agencies (State Forest Departments, ecological research institutions etc.). It is evident that regions with higher rate of reported strandings show heterogeneity in stranding frequencies. Based on EHSA, the south-eastern coast receives sporadic strandings despite multiple sections showing moderate to high stranding rates. On the other hand, consecutive and new hotspots emerge in the north-western region indicating need to assess the causes of increased stranding events.

Over one-third of the coastal districts reported < 5 strandings over the last ~ 270 years (highest number of districts in Gujarat, 7; followed by 5 districts in Kerala) indicating a lack of effort or reporting mechanism in the region. With data limitation arising from reporting bias, the patterns in the study emerge from a temporally disjunct dataset coming from various sources lacking homogeneity in the way information was obtained. The data was collected on an opportunistic basis rather than dedicated beach surveys, and thus the patterns obtained could be biased by factors which are likely to influence the behaviour of informants, such as the accessibility of the shore and weather conditions^70,71^ and unequal sampling effort^72^. In the Indian context, differences in data availability also arises with kind of marine mammal research undertaken, such as by fishery biologists in the past^47,73^ with focus in regions in proximity of the fishery institutions (such as Central Marine Fishery Research Institute), or other institutions (National Institute of Oceanography) or individual species or area focused efforts (e.g. along south Maharashtra coast by Konkan Cetacean Research Team on Indo-pacific humpback dolphins–^40,43,44,74^ or along dugong distribution sites by Wildlife Institute of India^42^ (CAMPA-Dugong Recovery Program—https://wii.gov.in/campa_Dugong). This is likely to influence the proportion of strandings of that species, which dominate the dataset, and patterns might not be generalizable or even applicable to the whole group as considered in the study, due to differences in their biology and behaviour. Similarly, the data received from the residents and fishers of any region is dependent on the motivation and awareness level regarding the importance of reporting strandings through social media or to curated online databases, or to the researchers working on marine mammals in the area.

Recently, the Ministry of Environment, Forest and Climate Change, Government of India released the Marine Megafauna Stranding Management Guidelines (https://pib.gov.in/PressReleasePage.aspx?PRID=1692990). This document is the first policy initiative from Government of India to manage marine mammal strandings in the country signalling recognition of this key conservation issue at apex governance level. These guidelines outline steps to handle live or dead strandings with recommendations for setting up a National Stranding Centre to coordinate stranding management with states. It recommends each state to set up a State Stranding Centre with directions to establish Local Stranding Networks and Rapid Response Teams to deal with future strandings. Further, it endorses formation of a National Stranding Database to collect and publish data received from State Stranding Centres through a website. These guidelines also highlight priority areas for bycatch reduction through outreach, training, and improvement in fishing practices.

Though these guidelines are a positive step forward to address gaps in stranding management, a robust framework is required to establish these proposed facilities at the national, state, and local level. It is important for states to adhere to these central guidelines in principle and not merely ‘report’ strandings but also intervene on mitigating the causes of strandings. The proposed National Stranding Centre (or National Marine Megafauna Stranding Centre) can be developed as a statutory body in the lines of National Tiger Conservation Authority to develop protocols, organise trainings, and support population assessments and monitoring exercises through State Forest Departments’ coordination, other allied ministries (Fisheries, Earth Sciences), and research groups. The release of these guidelines is timely with the announcement of Project Dolphin (https://pib.gov.in/PressReleasePage.aspx?PRID=1646491) by the Prime Minister of India. Substantial funding could be made available through this initiative for effective implementation of these guidelines. Further, establishment of local stranding networks have already been suggested in the India’s 3^rd^ National Wildlife Action Plan (https://wii.gov.in/nwap_2017_31). Standard stranding response protocols have proven to increase the efficiency of responding and recording of stranding events^75,76^, and protocols and training guidelines are readily and freely available from global resources (e.g., www.gmast.org). Periodic stranding response programs for training field veterinarians, frontline personnel of State Forest Department (especially around stranding hotspots identified through this study) would be crucial for successful implementation of these guidelines.

**Figure 7:**
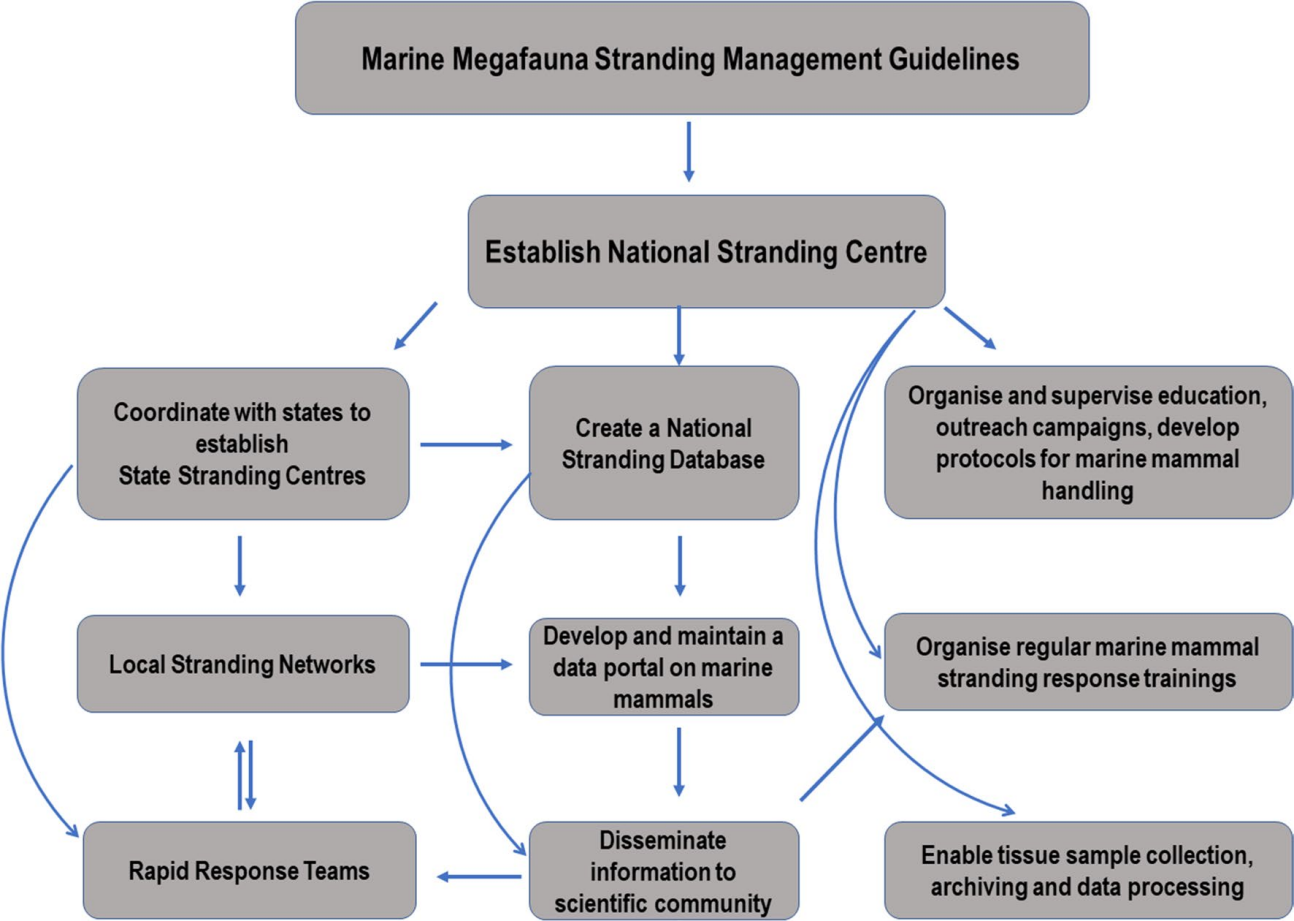
A framework to guide implementation of the Marine Megafauna Stranding Management Guidelines in India.

We suggest a framework (Fig. 7) for effective execution of the stranding management guidelines. This framework provides a direction to undertake measures required at central, state and organisation levels. The onus to implement the Marine Megafauna Stranding Management Guidelines lies with the Ministry of Environment, Forest and Climate Change and the Ministry should ideally be responsible for establishing a National Stranding Centre with adequate financial support (possibly from the proposed Project Dolphin program). The National Stranding Centre would advise the states to sequentially establish State Stranding Centres and Local Stranding Networks (prioritizing districts identified in the present study) enabled with Rapid Response Teams. Local Stranding Networks would feed data into a National Stranding Database operated by the National Stranding Centre. The centre should also supervise coastal surveillance to report strandings, recruit and train veterinary professionals in marine mammal-specific care, establish long-term monitoring (preferably with help from local NGOs, research institutions, independent researchers, university departments) at critical sites (Marine Protected Areas, Important Marine Mammal Areas) and organise trainings for handling marine mammal strandings for Rapid Response Teams and other volunteers/personnel. Additionally, the centre should develop protocols for marine mammal handling, rescue, and humane euthanasia for stranded animals in distress, coordinated response to unusual or unprecedented mass-stranding events, tissue archiving, sample processing and providing guidance to local stranding networks. These efforts can be disseminated through planned education and outreach campaigns involving a range of stakeholders from government agencies, Non-Governmental Organisations, to the public and local communities. The centre would enhance capacity of local stranding networks that enlists the volunteers from fishing community, public volunteers, sea turtle networks, and college students. Targeted support from fishing community for gathering stranding information could be achieved through incentivisation programs such as the Dugong Scholarship Scheme already in place in three states^77^. A cohesive coastal surveillance program that spans across taxa (marine mammals, sea turtles, seabirds, sharks, sea snakes) managed through the National Stranding Centre would help collect scientific data for assessing marine mega fauna populations across the country.

Marine mammals in India have received much less attention as compared to other species listed under the Wildlife (Protection) Act, 1972 of India. Our study shows that stranding data are a valuable tool to help formulate effective conservation strategies, guide national policies, and may serve as indicators of underlying change in source populations due to anthropogenic activities or natural events. We strongly believe that stranding hotspots identified in this study would provide an impetus to developing stranding response plans and hastening the process of setting up local stranding networks. These networks need to employ trained individuals, set up standardised publicly accessible databases and disseminate stranding response, including necropsy reports to the scientific and management community. A coordinated effort through the central agencies is required to enable data sharing with international entities and among researchers for ensuring long-term persistence of marine mammals in Indian waters.

## Methods

We compiled marine mammal strandings data available through a period of 269 years (1748 to 2017) on from various published and unpublished sources. The data sourcing, compilation, and analysis are described below.

- **Data sources**

We compiled marine mammal stranding records along India’s coasts from peer-reviewed publications, grey literature such as unpublished reports, thesis, newspaper articles and open access database (Table 3). We also collected data other than strandings (sightings, accidental net entanglements and hunting) to understand the species composition of marine mammals along Indian coastline and to give a comparative account of number of cases in each of these categories.

b. **Data compilation**

**Table 3:** Data sources, types of events and total number of records for each event for marine mammals in Indian waters compiled for a period from 1748—2017.

Data source	Strandings	Sightings	Incidental mortalities	Induced mortalities	Total records
Publication (n = 90) and doctoral thesis (n = 1)	225	70	139	6	440
Marine Mammal Research and Conservation Network of India (MMRCNI) database	287	102	80	17	486
Sivakumar and Nair, 2013	108	461	19	34	622
Database of the project ‘Recovery of Dugongs in India and their habitat: an integrated participatory approach’, Wildlife Institute of India	46	19	3	–	68
Newspaper articles reporting marine mammals	18	6	1	–	47 (including 22 unknown)

We compiled data on each record as provided by the author/ expert/ informant. We included information about the event (live or dead stranding/ sighting), species name (if identified), date, location, and any additional information (condition of carcass, age class, sex, and any other observations regarding the incident). We eliminated the records falling outside of Indian territorial waters (from Sri Lanka and Pakistan, received through MMRCNI database). Records from MMRCNI database found to be overlapping with publications (n = 168) were eliminated to avoid duplication.

We overlaid the coordinates of each event in the dataset on Google Earth Pro v.7.3 for visualization. In cases where exact coordinates were not available, the nearest point on the coast was marked manually. Records where only district information was available (n = 58), the approximate mid-point of the district coastline was marked for assigning the coordinates for spatial analysis.

c. **Data processing**

We split all records into four taxonomic groups:

- Baleen whales (BW) – consisting of all records of Family Balaenopteridae.
  Toothed whales were divided into two groups for the analysis purpose, taking into consideration the differences in behaviour:
- Sperm whales and beaked whales (SBW) consisting of toothed whale records belonging to Families Physeteridae and Ziphiidae.
- Dolphins and finless porpoise (DFP) consisting of members of Families Delphinidae and Phocoenidae.
- Dugongs– consisting of records of Family Dugongidae i.e., *Dugong dugon*.

This grouping ensured removal of biases arising from species misidentifications^70^ and assessing spatio-temporal patterns of the groups rather than individual species.

All the records were reclassified into following categories based on the information available in the source dataset:

- Sightings: records where live animals were sighted
- Incidental mortality: records where animals were found dead after entanglement in fishing nets or being struck by vessels
- Induced mortality: records where animals were reported hunted or killed or were driven ashore.
- Strandings: records where dead or live animals were found washed ashore, or floating near shore or stranded alive and were attempted for rescue
- Unknown: records where the state of the animal was not clearly mentioned by sources.

Further, the data was classified on the location of each record with respect to east or west coast of India. Records from the island archipelagos of Lakshadweep (LD) and Andaman & Nicobar Islands (ANI) were classified as islands records.

d. **Data Analysis**

The stranding records compiled above (n = 632) were used for both temporal and spatial analysis.

i. **Temporal analysis**

The stranding reports before 1975 were sporadic (n = 207 records spread over 227 years, from –1748 to 1975) and thus were excluded from further analysis. Initially, we investigated the changes in numbers of strandings across decades collectively for all groups. Later, to understand the differences in stranding numbers on a finer scale, we calculated the rate of reported strandings of each group by dividing the coastline into 50 km sections using ArcGIS 10.5 (details below) and assigned the total number of strandings to these sections. We obtained per kilometre stranding rates for each group for each 50 km section which were later pooled to compare the stranding rates of each group across both the coastlines.

Stranding rates were further used to visualise yearly and monthly patterns for each group separately. We used the Mann Whitney U test performed in R program to assess the significance of differences in the stranding rates of each group across the two coastlines. Stranding rates could not be calculated for Lakshadweep and Andaman & Nicobar Island archipelagos due to discontinuous coastlines.

ii. **Spatial analysis**

We divided the coastline of mainland India into 170 segments, each of 50 km length, using ‘Split’ tool in ArcGIS 10.5. Each stranding location was assigned to the nearest segment using the ‘Near Analysis’ tool. We calculated stranding rates per km (*s*_*r*_) for each of these segments by dividing the number of stranding events assigned to each segment by the length of the segment i.e. 50 km. Segments were classified manually as region of no (0 strandings per km), very low stranding events (0.01–0.02 strandings per km), low (0.03–0.08 strandings/km), medium (0.09 to 0.20 strandings/km) and high (> 0.21 strandings/km) rate of reported strandings.

iii. **Spatio-temporal analysis**

Space time cube

We used integrated spatial and temporal parameters of the data using the space–time cube tool of ArcGIS Pro v. 2.4.2. We used 15th of each month for the records which did not have precise date information (n = 62). Stranding locations were converted into a shape file and then imported into ArcGIS Pro version 2.4.2 using *Create Space Time Cube Using Aggregating Points* feature to generate patterns. This feature aggregates data points based on chosen spatial and temporal scale (ESRI ArcGIS, 2016) and uses Mann–Kendall statistics to identify trends ^76^. Mann–Kendall statistics is a rank correlation statistic between ranks of observations and their time sequence. It calculates the values for each bin and identifies the trend based on the z-score and p-value for each bin. A small p-value (< 0.05) indicates that the trend is statistically significant ^77^. We aggregated the strandings across each 50 km segment over the time-scale of 1-year period for the generation of space time cube. The spatial scale of 50 km was selected after testing various other sizes from 10 to 300 km. After comparing lengths of the coastal districts in India, the spatial scale of 50 km was finalised, which is the approximate length of the smallest coastal district in India.

Emerging hotspot analysis

We performed “Emerging Hotspot Analysis” with the NetCDF file created in space–time cube tool as an input for tool on ArcGIS Pro which performs Getis-Ord (Gi*) statistics for each bin of space time cube and the neighbouring bins^78^. The Gi* statistics calculates and compares the value of each bin in the space time cube to neighbouring bins and identifies hot spot trends based on the degree of association between two bins (ArcGIS, 2016; Getis and Ord, 1992). The software generates Z score (standard deviations) and P values (statistical probabilities) using the Gi* statistics for each bin, and these values are then compared with the neighbouring bins to determine the type of hotspot^79^. A Z score ≥ 1.96 or ≤ 1.96 signifies a statistically significant hotspot/ cold spot at a significance level of P < 0.05. As the stranding locations cluster in a bin, the Z score increases leading to a hotspot. Here, a hotspot is a section of the coastline with statistically significant clustering in both space and time.

The data was evaluated into eight types of hot and cold spot trends using Mann–Kendall statistics^78^. Gi* and Mann–Kendall statistics are then used to categorise each bin into 8 hot/cold spot patterns.

## Supplementary Information

Supplementary Information.
