# Supplementary material for: Spatio-temporal analysis identifies marine mammal stranding hotspots along the Indian coastline
Source: Sci Rep. 2022 Mar 8;12:4128. doi: 10.1038/s41598-022-06156-0 (PMC8904828; doi:10.1038/s41598-022-06156-0)
Supplement: Supplementary file 1 — Supplementary Information. [file 41598_2022_6156_MOESM1_ESM.docx]

**Supplementary material - Dudhat et al 2021**

This supplementary material reports in details the z-score and p-values for each grid of the Mann-Kendall trend test of the emerging hotspot analysis of marine mammal standings along Indian coastline. Numbers shown in the grids can be matched with the ID field in the corresponding tables for 1) all marine mammals, 2) baleen whales and 3) dolphins and finless porpoise groups respectively.


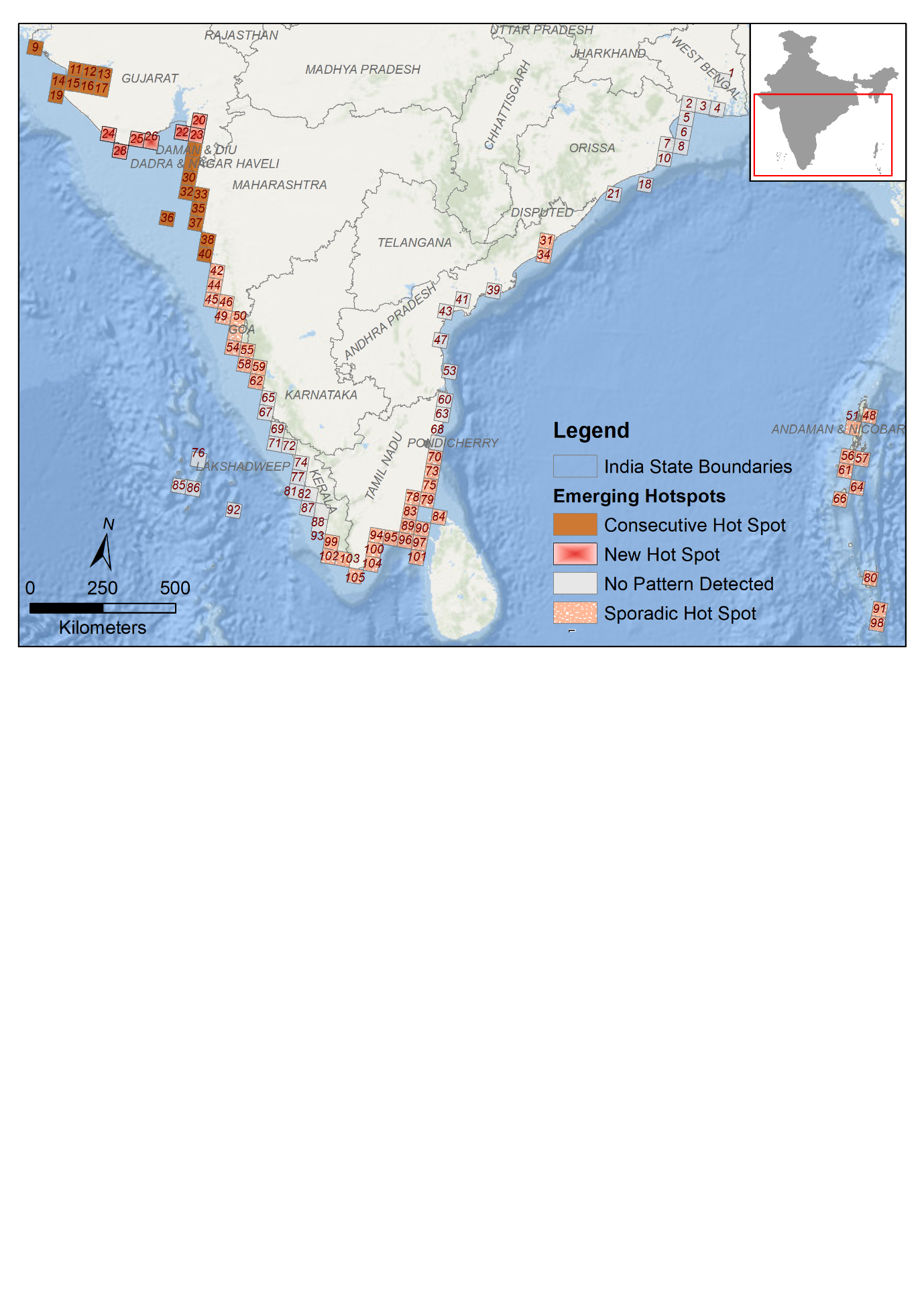


Supplementary Figure 1: Emerging hotspots for all stranded marine mammals along the Indian coastline. This figure supplements the information provided in Figure 6b. This map was created using ArcGIS Pro 2.4.2 (https://pro.arcgis.com/en/pro-app/2.8/tool-reference/space-time-pattern-mining/emerginghotspots.htm)

Supplementary Table 1: z-score and p-value of Mann-Kendall trend test for all marine mammals stranded along the Indian coastline.

| **Grid ID** | **Pattern type** | **Z score** | **p value** |
| --- | --- | --- | --- |
| 1, 2, 3, 4, 5, 6 | No Pattern Detected | 3.950148 | 0.000078 |
| 7, 8 | No Pattern Detected | 4.531043 | 0.000006 |
| 9 | Consecutive Hot Spot | 5.880945 | 0.000000 |
| 10 | No Pattern Detected | 5.081902 | 0.000000 |
| 11, 12 | Consecutive Hot Spot | 5.880945 | 0.000000 |
| 13 | Consecutive Hot Spot | 5.554528 | 0.000000 |
| 14, 15 | Consecutive Hot Spot | 5.880945 | 0.000000 |
| 16, 17 | Consecutive Hot Spot | 5.554528 | 0.000000 |
| 18 | No Pattern Detected | 5.081902 | 0.000000 |
| 19 | Consecutive Hot Spot | 5.880945 | 0.000000 |
| 20 | New Hot Spot | 3.101349 | 0.001926 |
| 21 | No Pattern Detected | 5.308591 | 0.000000 |
| 22, 23 | New Hot Spot | 3.101349 | 0.001926 |
| 24 | New Hot Spot | 5.553885 | 0.000000 |
| 25 | New Hot Spot | 4.550510 | 0.000005 |
| 26 | New Hot Spot | 4.241472 | 0.000022 |
| 27 | Consecutive Hot Spot | 3.467935 | 0.000524 |
| 28 | New Hot Spot | 4.268400 | 0.000020 |
| 29 | Consecutive Hot Spot | 4.261754 | 0.000020 |
| 30 | Consecutive Hot Spot | 4.292407 | 0.000018 |
| 31 | Sporadic Hot Spot | 4.052493 | 0.000051 |
| 32, 33 | Consecutive Hot Spot | 4.846128 | 0.000001 |
| 34 | Sporadic Hot Spot | 3.964837 | 0.000073 |
| 35, 36 | Consecutive Hot Spot | 4.365663 | 0.000013 |
| 37 | Consecutive Hot Spot | 4.593999 | 0.000004 |
| 38 | Consecutive Hot Spot | 4.876845 | 0.000001 |
| 39 | No Pattern Detected | 6.063126 | 0.000000 |
| 40 | Consecutive Hot Spot | 4.819058 | 0.000001 |
| 41 | No Pattern Detected | 6.063126 | 0.000000 |
| 42 | Sporadic Hot Spot | 4.078283 | 0.000045 |
| 43 | No Pattern Detected | 6.063126 | 0.000000 |
| 44 | Sporadic Hot Spot | 3.836949 | 0.000125 |
| 45 | Sporadic Hot Spot | 3.836904 | 0.000125 |
| 46 | Sporadic Hot Spot | 6.029426 | 0.000000 |
| 47 | No Pattern Detected | 6.115006 | 0.000000 |
| 48 | Sporadic Hot Spot | 4.398028 | 0.000011 |
| 49 | Sporadic Hot Spot | 6.029426 | 0.000000 |
| 50 | Sporadic Hot Spot | 6.277881 | 0.000000 |
| 51 | Sporadic Hot Spot | 4.398028 | 0.000011 |
| 52 | Sporadic Hot Spot | 6.898166 | 0.000000 |
| 53 | No Pattern Detected | 6.115006 | 0.000000 |
| 54, 55 | Sporadic Hot Spot | 7.299691 | 0.000000 |
| 56, 57 | Sporadic Hot Spot | 4.398028 | 0.000011 |
| 58 | Sporadic Hot Spot | 7.760016 | 0.000000 |
| 59 | Sporadic Hot Spot | 7.938597 | 0.000000 |
| 60 | No Pattern Detected | 7.168471 | 0.000000 |
| 61 | Sporadic Hot Spot | 4.398028 | 0.000011 |
| 62 | Sporadic Hot Spot | 8.031237 | 0.000000 |
| 63 | No Pattern Detected | 7.146853 | 0.000000 |
| 64 | Sporadic Hot Spot | 4.398028 | 0.000011 |
| 65 | No Pattern Detected | 7.853948 | 0.000000 |
| 66 | Sporadic Hot Spot | 4.398028 | 0.000011 |
| 67 | No Pattern Detected | 7.058627 | 0.000000 |
| 68 | No Pattern Detected | 7.665645 | 0.000000 |
| 69 | No Pattern Detected | 6.622229 | 0.000000 |
| 70 | Sporadic Hot Spot | 7.849621 | 0.000000 |
| 71, 72 | No Pattern Detected | 5.909952 | 0.000000 |
| 73 | Sporadic Hot Spot | 8.911070 | 0.000000 |
| 74 | No Pattern Detected | 4.612047 | 0.000004 |
| 75 | Sporadic Hot Spot | 8.911070 | 0.000000 |
| 76 | No Pattern Detected | 5.841414 | 0.000000 |
| 77 | No Pattern Detected | 4.882456 | 0.000001 |
| 78 | Sporadic Hot Spot | 9.862511 | 0.000000 |
| 79 | Sporadic Hot Spot | 9.849749 | 0.000000 |
| 80 | Sporadic Hot Spot | 4.394935 | 0.000011 |
| 81 | No Pattern Detected | 4.882456 | 0.000001 |
| 82 | No Pattern Detected | 5.394446 | 0.000000 |
| 83 | Sporadic Hot Spot | 9.916867 | 0.000000 |
| 84 | Sporadic Hot Spot | 9.955519 | 0.000000 |
| 85, 86 | No Pattern Detected | 4.028607 | 0.000056 |
| 87 | No Pattern Detected | 5.031844 | 0.000000 |
| 88 | No Pattern Detected | 3.483522 | 0.000495 |
| 89 | Sporadic Hot Spot | 9.346976 | 0.000000 |
| 90 | Sporadic Hot Spot | 9.118926 | 0.000000 |
| 91 | Sporadic Hot Spot | 4.394935 | 0.000011 |
| 92 | No Pattern Detected | 4.643434 | 0.000003 |
| 93 | No Pattern Detected | 3.483522 | 0.000495 |
| 94 | Sporadic Hot Spot | 8.089532 | 0.000000 |
| 95 | Sporadic Hot Spot | 8.803225 | 0.000000 |
| 96 | Sporadic Hot Spot | 9.089505 | 0.000000 |
| 97 | Sporadic Hot Spot | 9.118926 | 0.000000 |
| 98 | Sporadic Hot Spot | 4.394935 | 0.000011 |
| 99 | Sporadic Hot Spot | 5.021299 | 0.000001 |
| 100 | Sporadic Hot Spot | 7.769016 | 0.000000 |
| 101 | Sporadic Hot Spot | 9.347130 | 0.000000 |
| 102 | Sporadic Hot Spot | 5.825356 | 0.000000 |
| 103 | Sporadic Hot Spot | 5.822841 | 0.000000 |
| 104, 105 | Sporadic Hot Spot | 6.310982 | 0.000000 |


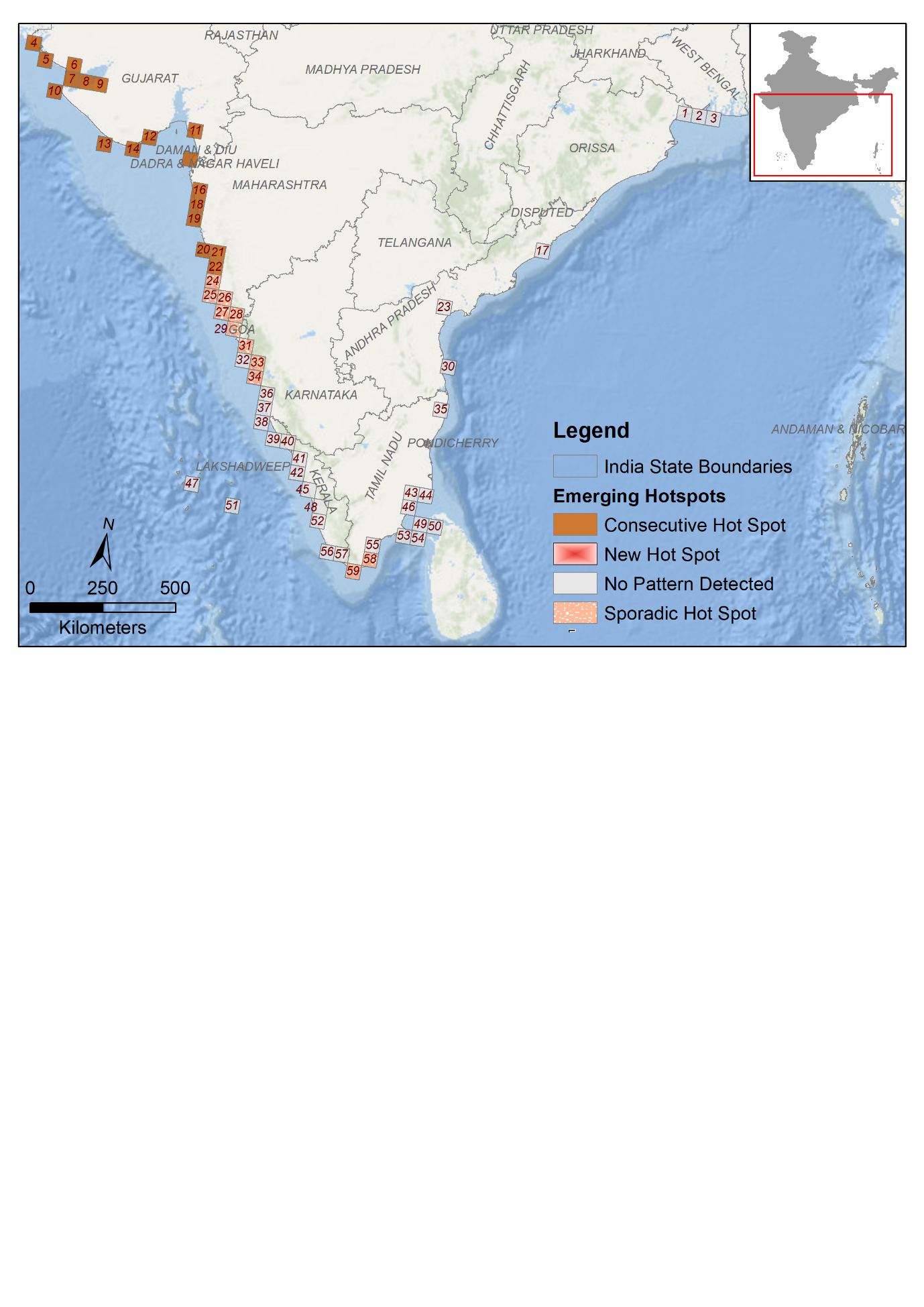


Supplementary Figure 2: Emerging hotspots for all stranded baleen whales stranded along the Indian coastline. This figure supplements the information provided in Figure 6c. This map was created using ArcGIS Pro 2.4.2 (https://pro.arcgis.com/en/pro-app/2.8/tool-reference/space-time-pattern-mining/emerginghotspots.htm)

Supplementary Table 2: z-score and p-value of Mann-Kendall trend test for baleen whales stranded along the Indian coastline.

| **Grid ID** | **Pattern type** | **Z score** | **p value** |
| --- | --- | --- | --- |
| 1, 2, 3 | No Pattern Detected | 3.997391 | 0.000064 |
| 4, 5, 6, 7, 8 | Consecutive Hot Spot | 1.110855 | 0.266631 |
| 9 | Consecutive Hot Spot | 1.073980 | 0.282832 |
| 10 | Consecutive Hot Spot | 1.110855 | 0.266631 |
| 11 | Consecutive Hot Spot | 1.836480 | 0.066287 |
| 12 | Consecutive Hot Spot | 1.710079 | 0.087251 |
| 13 | Consecutive Hot Spot | 1.063796 | 0.287421 |
| 14 | Consecutive Hot Spot | 1.710079 | 0.087251 |
| 15, 16 | Consecutive Hot Spot | 1.279716 | 0.200645 |
| 17 | No Pattern Detected | 3.997391 | 0.000064 |
| 18 | Consecutive Hot Spot | 0.754810 | 0.450363 |
| 19 | Consecutive Hot Spot | 0.870195 | 0.384194 |
| 20 | Consecutive Hot Spot | 0.960714 | 0.336696 |
| 21 | Consecutive Hot Spot | 1.653462 | 0.098237 |
| 22 | Consecutive Hot Spot | 1.046345 | 0.295402 |
| 23 | No Pattern Detected | 5.406543 | 0.000000 |
| 24, 25 | Sporadic Hot Spot | 0.406543 | 0.684343 |
| 26 | Sporadic Hot Spot | 1.355348 | 0.175307 |
| 27, 28 | Sporadic Hot Spot | 1.710939 | 0.087092 |
| 29 | Sporadic Hot Spot | 4.296188 | 0.000017 |
| 30 | No Pattern Detected | 3.982869 | 0.000068 |
| 31 | Sporadic Hot Spot | 4.614061 | 0.000004 |
| 32 | No Pattern Detected | 4.224408 | 0.000024 |
| 33 | Sporadic Hot Spot | 4.175544 | 0.000030 |
| 34 | Sporadic Hot Spot | 3.630238 | 0.000283 |
| 35 | No Pattern Detected | 4.614390 | 0.000004 |
| 36 | No Pattern Detected | 4.119067 | 0.000038 |
| 37 | No Pattern Detected | 3.244161 | 0.001178 |
| 38 | No Pattern Detected | 2.741043 | 0.006124 |
| 39 | No Pattern Detected | 2.740379 | 0.006137 |
| 40 | No Pattern Detected | 2.494379 | 0.012618 |
| 41, 42 | No Pattern Detected | 1.715077 | 0.086331 |
| 43, 44 | No Pattern Detected | 5.199348 | 0.000000 |
| 45 | No Pattern Detected | 1.118214 | 0.263475 |
| 46 | No Pattern Detected | 5.199348 | 0.000000 |
| 47 | No Pattern Detected | 2.040239 | 0.041327 |
| 48 | No Pattern Detected | 1.832083 | 0.066939 |
| 49, 50 | No Pattern Detected | 5.199348 | 0.000000 |
| 51 | No Pattern Detected | 2.363108 | 0.018122 |
| 52 | No Pattern Detected | 2.594412 | 0.009475 |
| 53, 54 | No Pattern Detected | 5.199348 | 0.000000 |
| 55 | No Pattern Detected | 3.736894 | 0.000186 |
| 56, 57 | No Pattern Detected | 3.138566 | 0.001698 |
| 58 | Sporadic Hot Spot | 3.748301 | 0.000178 |
| 59 | Sporadic Hot Spot | 3.887336 | 0.000101 |


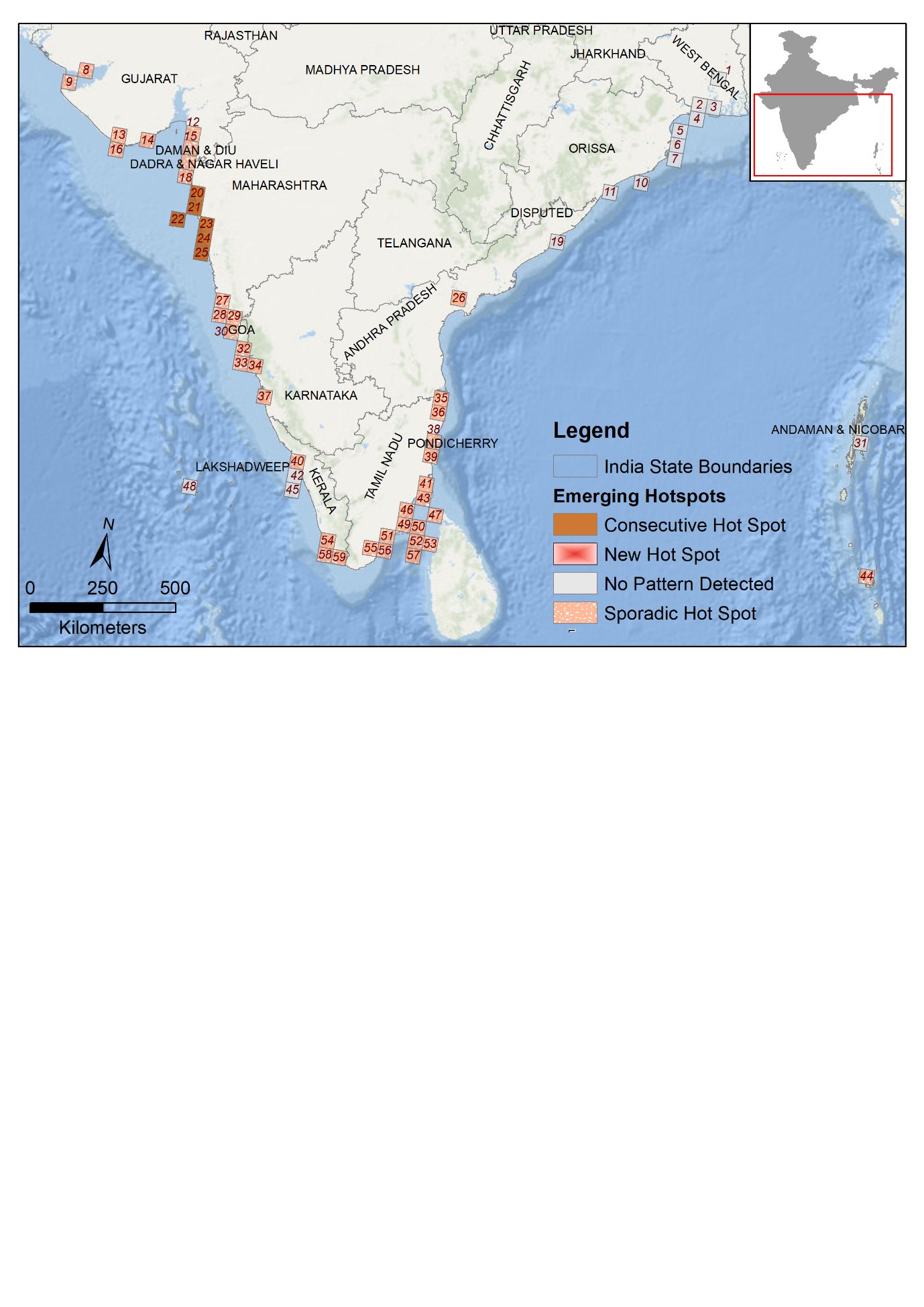


Supplementary Figure 3: Map showing emerging hotspots for all dolphins and finless porpoise stranded along the Indian coastline. This figure supplements the information provided in Figure 6d. This map was created using ArcGIS Pro 2.4.2 (https://pro.arcgis.com/en/pro-app/2.8/tool-reference/space-time-pattern-mining/emerginghotspots.htm)

Supplementary Table 3: z-score and p-value of Mann-Kendall trend test for small cetaceans stranded along the Indian coastline.

| **Grid ID** | **Pattern type** | **Z score** | **p value** |
| --- | --- | --- | --- |
| 1, 2, 3, 4, 5, 6, 7 | No Pattern Detected | 5.156363 | 0.000000 |
| 8, 9 | Sporadic Hot Spot | 3.286114 | 0.001016 |
| 10, 11 | No Pattern Detected | 6.090311 | 0.000000 |
| 12 | Sporadic Hot Spot | 3.170095 | 0.001524 |
| 13 | Sporadic Hot Spot | 3.286114 | 0.001016 |
| 14 | Sporadic Hot Spot | 3.170095 | 0.001524 |
| 15 | Sporadic Hot Spot | 3.763414 | 0.000168 |
| 16 | Sporadic Hot Spot | 3.286114 | 0.001016 |
| 17, 18 | Sporadic Hot Spot | 3.864672 | 0.000111 |
| 19 | No Pattern Detected | 6.646188 | 0.000000 |
| 20, 21, 22, 23 | Consecutive Hot Spot | 3.893908 | 0.000099 |
| 24 | Consecutive Hot Spot | 3.615474 | 0.000300 |
| 25 | Consecutive Hot Spot | 4.417788 | 0.000010 |
| 26 | Sporadic Hot Spot | 7.154768 | 0.000000 |
| 27, 28 | Sporadic Hot Spot | 4.566586 | 0.000005 |
| 29, 30 | Sporadic Hot Spot | 4.571309 | 0.000005 |
| 31 | No Pattern Detected | 6.616352 | 0.000000 |
| 32 | Sporadic Hot Spot | 4.571309 | 0.000005 |
| 33, 34 | Sporadic Hot Spot | 5.046675 | 0.000000 |
| 35, 36 | Sporadic Hot Spot | 6.424229 | 0.000000 |
| 37 | Sporadic Hot Spot | 5.046675 | 0.000000 |
| 38, 39 | Sporadic Hot Spot | 6.836135 | 0.000000 |
| 40 | Sporadic Hot Spot | 1.945872 | 0.051670 |
| 41 | Sporadic Hot Spot | 5.948701 | 0.000000 |
| 42 | No Pattern Detected | 2.112115 | 0.034677 |
| 43 | Sporadic Hot Spot | 6.058179 | 0.000000 |
| 44 | Sporadic Hot Spot | 5.701858 | 0.000000 |
| 45 | No Pattern Detected | 2.112115 | 0.034677 |
| 46 | Sporadic Hot Spot | 6.045782 | 0.000000 |
| 47 | Sporadic Hot Spot | 6.084464 | 0.000000 |
| 48 | No Pattern Detected | 3.914704 | 0.000091 |
| 49 | Sporadic Hot Spot | 5.579081 | 0.000000 |
| 50 | Sporadic Hot Spot | 5.622241 | 0.000000 |
| 51 | Sporadic Hot Spot | 3.776001 | 0.000159 |
| 52, 53 | Sporadic Hot Spot | 4.844467 | 0.000001 |
| 54 | Sporadic Hot Spot | 3.079704 | 0.002072 |
| 55 | Sporadic Hot Spot | 2.861231 | 0.004220 |
| 56 | Sporadic Hot Spot | 2.994007 | 0.002753 |
| 57 | Sporadic Hot Spot | 4.013506 | 0.000060 |
| 58 | Sporadic Hot Spot | 3.079704 | 0.002072 |
| 59 | Sporadic Hot Spot | 2.861231 | 0.004220 |
